# *Crif1* Deficiency Reduces Adipose OXPHOS Capacity and Triggers Inflammation and Insulin Resistance in Mice

**DOI:** 10.1371/journal.pgen.1003356

**Published:** 2013-03-14

**Authors:** Min Jeong Ryu, Soung Jung Kim, Yong Kyung Kim, Min Jeong Choi, Surendar Tadi, Min Hee Lee, Seong Eun Lee, Hyo Kyun Chung, Saet Byel Jung, Hyun-Jin Kim, Young Suk Jo, Koon Soon Kim, Sang-Hee Lee, Jin Man Kim, Gi Ryang Kweon, Ki Cheol Park, Jung Uee Lee, Young Yun Kong, Chul-Ho Lee, Jongkyeong Chung, Minho Shong

**Affiliations:** 1 Research Center for Endocrine and Metabolic Diseases, Chungnam National University School of Medicine, Daejeon, Korea; 2 Department of Pathology, Chungnam National University School of Medicine, Daejeon, Korea; 3 Department of Biochemistry, Chungnam National University School of Medicine, Daejeon, Korea; 4 Department of Pathology, Daejeon St. Mary's Hospital, The Catholic University of Korea, Daejeon, Korea; 5 School of Biological Sciences, Seoul National University, Seoul, Korea; 6 Animal Model Center, Korea Research Institute of Bioscience and Biotechnology, Daejeon, Korea; Ecole Polytechnique Fédérale de Lausanne, Switzerland

## Abstract

Impaired mitochondrial oxidative phosphorylation (OXPHOS) has been proposed as an etiological mechanism underlying insulin resistance. However, the initiating organ of OXPHOS dysfunction during the development of systemic insulin resistance has yet to be identified. To determine whether adipose OXPHOS deficiency plays an etiological role in systemic insulin resistance, the metabolic phenotype of mice with OXPHOS–deficient adipose tissue was examined. Crif1 is a protein required for the intramitochondrial production of mtDNA–encoded OXPHOS subunits; therefore, *Crif1* haploinsufficient deficiency in mice results in a mild, but specific, failure of OXPHOS capacity *in vivo*. Although adipose-specific *Crif1*-haploinsufficient mice showed normal growth and development, they became insulin-resistant. *Crif1*-silenced adipocytes showed higher expression of chemokines, the expression of which is dependent upon stress kinases and antioxidant. Accordingly, examination of adipose tissue from *Crif1*-haploinsufficient mice revealed increased secretion of MCP1 and TNFα, as well as marked infiltration by macrophages. These findings indicate that the OXPHOS status of adipose tissue determines its metabolic and inflammatory responses, and may cause systemic inflammation and insulin resistance.

## Author Summary

Type 2 diabetes is one of the most challenging health problems in the 21^st^ century. Although insulin resistance is regarded as a fundamental defect that precedes the development of type 2 diabetes, the nature and cause of insulin resistance remain unknown. Adipose tissue is an important organ that determines whole-body energy metabolism, and its dysfunction is a critical element in the development of systemic insulin resistance. Adipose mitochondrial function is suppressed in the insulin-resistant state, and increased adipose mitochondrial biogenesis is associated with the reversal of insulin resistance by a PPARγ agonist. However, despite these important observations, little is known about how mitochondrial respiratory dysfunction in white adipose tissue (WAT) causes insulin resistance. To determine whether adipose deficiency of mitochondrial respiratory capacity plays an etiological role in systemic insulin resistance, the metabolic phenotype of mice with mitochondrial OXPHOS (oxidative phosphorylation)–deficient adipose tissue was examined. Crif1 is a protein required for the translation of mtDNA–encoded OXPHOS subunits. Interestingly, mice haploinsufficient for *Crif1* in adipose tissue showed reduced OXPHOS capacity and developed marked insulin resistance.

## Introduction

White adipose tissue (WAT) determines whole-body energy metabolism by controlling lipid storage and by releasing adipokines, which may directly or indirectly affect the physiological functions of almost all cell types (for a review, see [1], [2]). These adipocyte functions are perturbed by genetic and environmental factors, which lead to adipocyte dysfunction characterized by hypertrophy, hypoxia and inflammatory process within adipose tissue [3]. Adipocyte dysfunction is further characterized by impaired insulin sensitivity, which is associated with changes in cellular composition or organelle dysfunction, particularly of the endoplasmic reticulum (ER) and mitochondria. An emerging concept to explain insulin resistance in obese individuals is maladaptive responses within the ER, which are prominent in adipose tissue (for a review, see [4]–[6]).

Besides the ER, the mitochondria in white adipocytes are linked with adipocyte differentiation and with the function of mature adipocytes. Recent studies show that drastic increases in mitochondrial biogenesis and reactive oxygen species (ROS) production via the OXPHOS complex play a crucial role in adipocyte differentiation. In addition, the mitochondria in differentiating adipocytes support high energy-consuming lipogenic processes to maintain mature adipocyte function [5], [7]. Therefore, it is suggested that the contribution of adipocyte mitochondria to whole-body energy metabolism or adipocyte plasticity may depend on the mitochondrial OXPHOS capacity of the adipose tissue [6]. Consistent with this, decreased mitochondrial capacity in adipocytes may also alter their insulin sensitivity and/or function due to the high energy requirements of fatty acid storage, adipokine secretion, insulin signaling, and glucose uptake [8], [9].

It is interesting that a marked decrease in the level of transcripts for nuclear-encoded mitochondrial genes in cells derived from the epididymal fat pads of *ob/ob* mice accompanies the onset of obesity [10]. In *db/db* and diet-induced obese mice, the expression of OXPHOS genes was markedly reduced compared with that in *db*/+ mice and control mice fed a standard-fat diet, respectively [11]. In humans, the mtDNA copy number is enriched in adipocytes in adipose tissue, but it decreases slightly with age and increasing BMI, and shows a strong positive correlation with basal and insulin-stimulated lipogenesis in fat cells [12]. More interestingly, suppression of OXPHOS genes is prominent in the visceral adipose tissue of humans with type 2 diabetes independent of obesity [13]. Agonists of peroxisome proliferator-activated receptor-gamma (PPARγ) increase the number of mitochondria and induce mitochondrial remodeling in adipocytes [10], [11], [14], and significantly increase the mitochondrial copy number and expression of factors involved in mitochondrial biogenesis, including PPARγ coactivator-1alpha (PGC1α) and mitochondrial transcription factor A (TFAM), which are required for mitochondrial transcription of OXPHOS genes in humans [15]. These observations in rodent models and human subjects suggest that the OXPHOS capacity of adipose tissue may affect the changes in adipocyte plasticity, which controls insulin sensitivity and may determine the therapeutic responsiveness to antidiabetic agents such as thiazolidinediones and CB1 receptor blockers that affect the mitochondrial content of adipocytes [10], [16].

Here, we demonstrate that primary OXPHOS dysfunction in adipose tissue causes insulin resistance and a diabetic phenotype in mice with a *Crif1* loss-of-function mutation. Crif1 is a mitochondrial protein that associates with large mitoribosomal subunits, which are located close to the polypeptide exit tunnel, and the elimination of *Crif1* led to both aberrant synthesis and defective insertion of mtDNA-encoded nascent OXPHOS polypeptides into the inner membrane [17]. Targeted elimination of the *Crif1* gene resulted in a phenotype characterized by organ-specific failure of OXPHOS function; therefore, we attempted to identify the adipose tissue phenotypes of adipose-specific *Crif1*-knockout mice using Fabp4-*Cre* and Adiponectin-*Cre* mice models. Reduced OXPHOS capacity in the WAT of *Crif1*-deficient mice triggered spontaneous adipose inflammation, which was characterized by macrophage infiltration and systemic insulin resistance. Therefore, the OXPHOS reserve may be the critical determinant controlling the metabolic and inflammatory responses of adipose tissue, which are closely related to systemic changes in insulin sensitivity.

## Results

### Homozygotic loss of *Crif1* causes marked impairment of WAT development

Crif1 is a mitochondrial protein that specifically interacts with the protein components of the large subunit of the mitochondrial ribosome [17]. It specifically regulates the translation and insertion of the 13 polypeptide subunits that comprise mitochondrial OXPHOS complexes I, III, IV and V. Homozygous *Crif1*-null mouse embryonic fibroblasts (MEFs) showed a profound failure in translation and expression of these subunits, along with markedly low levels of basal and stimulated (CCCP-treated) mitochondrial oxygen consumption [17]. Disruption of the mouse *Crif1* gene consistently resulted in a profound OXPHOS deficiency characterized by the loss of OXPHOS complex subunits and respiratory complexes *in vivo*.

*Crif1* mRNA is ubiquitously expressed, and it is highly expressed in brain, heart, liver kidney and skeletal muscle (Figure S1A). Two types of adipose tissues, brown (BAT) and white (WAT), contained substantial amounts of *Crif1* mRNA (Figure S1A). *Crif1* mRNA levels were decreased in the WAT, BAT and liver of *db/db* and *ob/ob* mice compared to *db/+* and *ob/+* mice, respectively (Figure S1B). Interestingly, *Crif1* mRNA expression in WAT of C57BL/6 mice was downregulated when they were fed a high fat diet (HFD) for 8 weeks (Figure S1C). These findings indicate that *Crif1* expression correlates with the nutritional status in adipose tissue.

**Figure 1:**
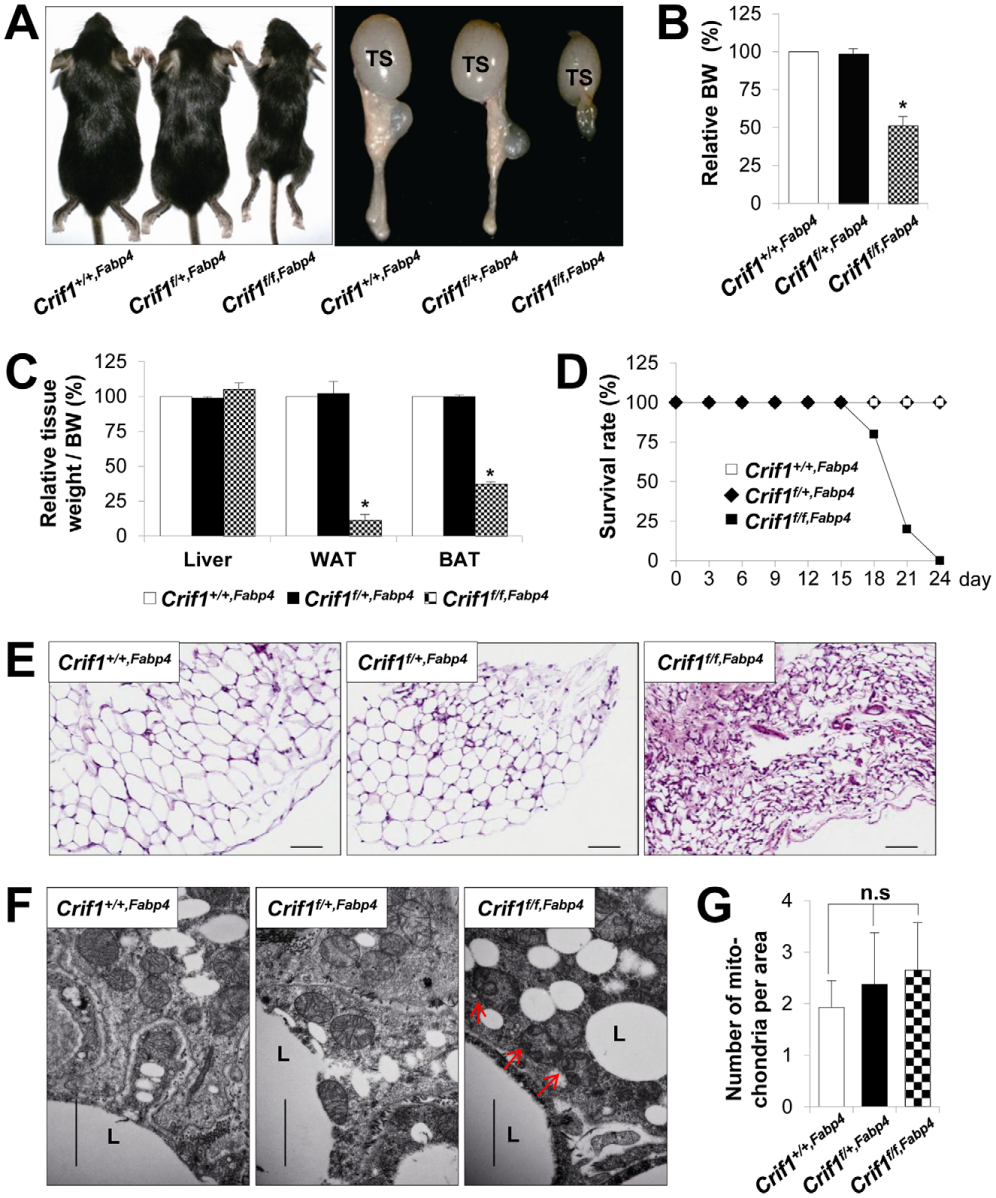
Marked failure of adipose tissue development in *Crif1^f/f,Fabp4^* mice. Mice were generated by breeding two mouse lines, a *Crif1^flox/flox^* transgenic mouse line and a Fabp4-*Cre* recombinase transgenic mouse line. Data from *Crif1^f/f,Fabp4^* mice were obtained at 3 weeks-of-age. (A) Gross characteristics of control mice (*Crif1^+/+,Fabp4^*), adipose-specific *Crif1* heterozygous mice (*Crif1^f/+,Fabp4^*), and adipose-specific *Crif1* homozygous knockout mice (*Crif1^f/f,Fabp4^*). TS, testis. (B and C) Body weight and tissue weight relative to control mice (*n* = 10). Values are means + SD, *p<0.05 versus control mice. BAT, brown adipose tissue; WAT, white adipose tissue. (D) *Crif1^f/f,Fabp4^* mice showed markedly reduced survival rates after 2 weeks-of-age (*n* = 20). (E) Hematoxylin & eosin (H&E) staining of perirenal WAT (pWAT). Scale: 100 µm. (F) Transmission electron microscopy (TEM) of subcutaneous WAT (sWAT) revealed that the mitochondria of *Crif1^f/f,Fabp4^* mice developed swollen cristae (red arrows). L, lipid droplet. Scale: 6,000 nm. (G) Mitochondria number per area in sWAT (*n* = 10). Values are means + SD. n.s, not significant.

To identify the roles of *Crif1* and mitochondrial OXPHOS in adipose tissue, we tried to induce primary OXPHOS deficiency in adipose tissue *in vivo* using conditional *Crif1* knockout mice. We crossed conditional *Crif1* mice (*Crif1^flox/flox^*) [18] with mice expressing a *Cre* recombinase gene under the control of the fatty acid binding protein-4 (Fabp4) promoter (Fabp4-*Cre*) and the adiponectin promoter (Adipoq-*Cre*). The resulting pups were born healthy and viable, and showed a normal Mendelian ratio. However, these homozygous *Crif1^f/f,Fabp4^* mice showed delayed weight gain and poor development of adipose tissue (Figure 1A–1C). Unlike the control (*Crif1^+/+,Fabp4^*) and *Crif1* heterozygous (*Crif1^f/+Fabp4^*) mice, *Crif1^f/f,Fabp4^* mice showed uniform lethality within 24 days of birth (median survival = 19.4 days) (Figure 1D).

The perirenal, subcutaneous and epididymal fat pads of *Crif1^f/f,Fabp4^* mice comprised small adipocytes with dystrophic changes (Figure 1E). To verify any mitochondrial abnormalities, the adipose tissues of *Crif1^f/f,Fabp4^* mice were examined by transmission electron microscopy (TEM). The adipocytes of these mice contained mitochondria with ultrastructural abnormalities, such as swollen and distorted cristae, but mitochondrial number was unaffected (Figure 1F and 1G). In heterozygous *Crif1^f/+,Fabp4^* mice, hematoxylin and eosin (H&E) staining of adipose tissue showed no evidence of histological abnormalities compared with the controls (Figure 1E). Consistent with the results of H&E staining, the mitochondria of *Crif1^f/+,Fabp4^* mice showed no morphological or numerical abnormalities of mitochondria in TEM (Figure 1F and 1G). Collectively, this comprehensive analysis of the adipose tissues in *Crif1^f/f,Fabp4^* mice indicated that loss of *Crif1* results in a marked failure of WAT and BAT development.

### Characterization of BAT in *Crif1^f/f,Fabp4^* mice

The Fabp4-*Cre* transgene is expressed and localized within the dorsal root ganglion, centrum of the vertebra and the carpals of the embryo from the mid-gestation stage [19]. Neonatal *Crif1^f/+,Fabp4^* or *Crif1^f/f,Fabp4^* mice did not show developmental abnormalities when compared with control mice. Therefore, embryonic expression of the Fabp4-*Cre* transgene may not affect the development of *Crif1^f/+,Fabp4^* and *Crif1^f/f,Fabp4^* mice, and may not be a plausible reason for observed lethality at around post-natal Week 3. Mice are normally weaned at post-natal Week 3, at which point the rate of lipogenesis and UCP1 expression in the BAT rises sharply and reaches maximal levels to enhance thermogenesis [20]. The Fabp4-*Cre* transgene was uniformly detected in BAT from the early post-natal period (Day 7), the Crif1 protein and OXPHOS complex subunits are downregulated in the BAT of 3-week-old mice (Table S1). As shown in Figure 1C, *Crif1^f/f,Fabp4^* mice had less BAT at Day 21, but histological examination of inter-scapular BAT showed normal histological findings (Figure S2A). *Crif1^f/f,Fabp4^* mice had fewer mitochondria than control mice, but these were larger in size and were characteristically disorganized and swollen, suggesting OXPHOS defects (Figure S2B and S2C). Consistent with these findings, *Crif1^f/f,Fabp4^* mice showed a low body temperature under ambient conditions (23°C) and rapidly reached a fatally low rectal temperature within 5 minutes of immersion in cold water (4°C) (Figure S2D). However, although the mass of BAT was reduced, the level of UCP1 expression was not altered in *Crif1^f/f,Fabp4^* mice (data not shown). When *Crif1^f/f,Fabp4^* mice were housed at thermoneutrality (30°C), the median survival rate was increased and mortality was reduced (Figure S2E). This indicates that thermal stress caused by mitochondrial OXPHOS dysfunction in BAT following *Crif1* ablation may be a critical factor in the premature death of *Crif1^f/f,Fabp4^* mice.

By contrast, the BAT of *Crif1^f/+,Fabp4^* mice showed normal development and histological and ultrastructural findings (Figure S2A–S2C). Furthermore, the response of *Crif1^f/+,Fabp4^* mice (in terms of core temperature) to a cold environment were identical to those of control mice (Figure S2D). These results showed that Fabp4-*Cre* driven haploinsufficiency of *Crif1* may not affect the physiological function of BAT.

### Characterization of mitochondrial OXPHOS function in *Crif1^f/+,Fabp4^* mice

**Figure 2:**
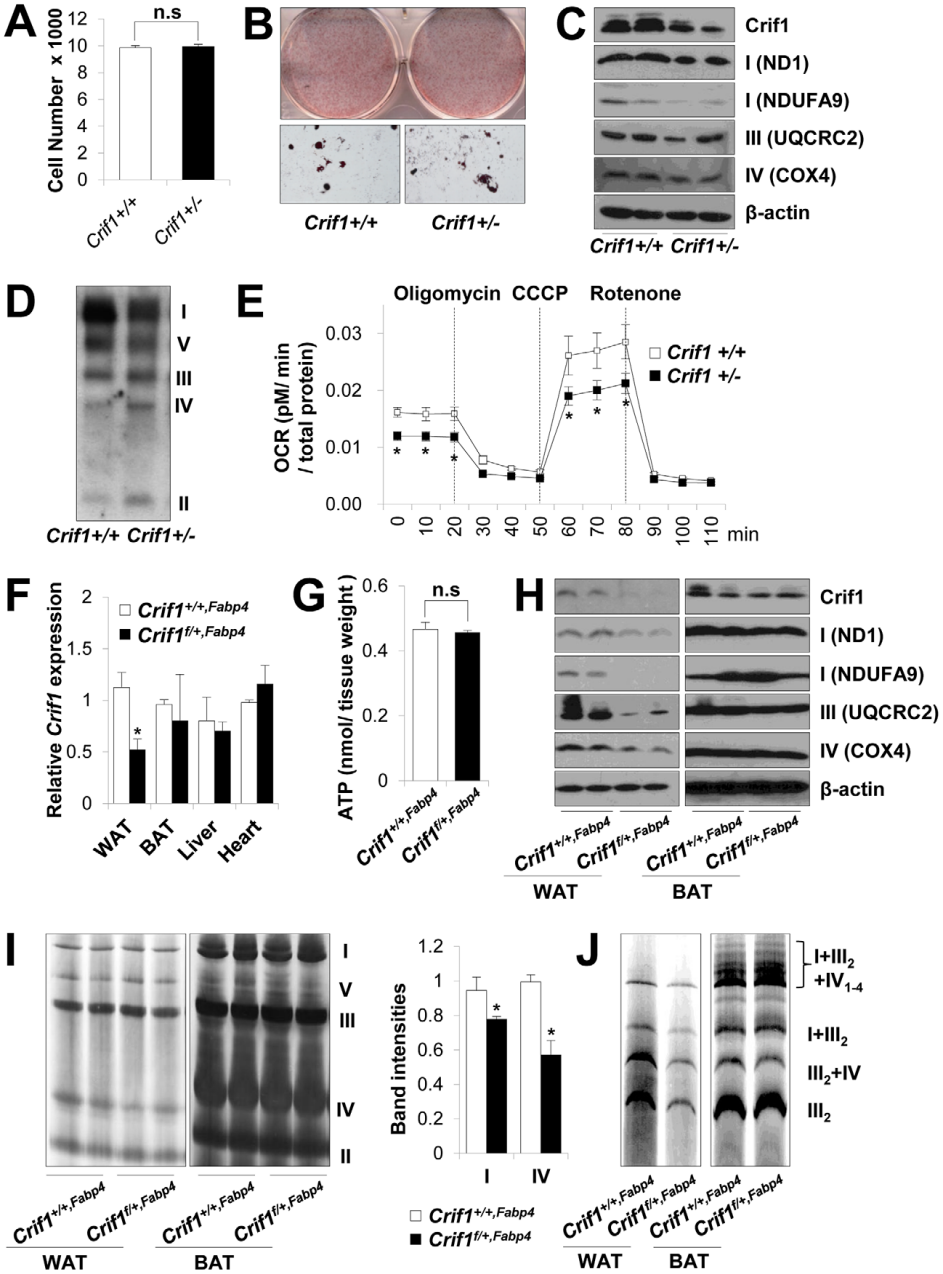
Dysfunctional mitochondria in *Crif1* haploinsufficient adipocytes. (A) Adipose derived stem cells (ADSCs) were isolated from eWAT of *Crif1^+/+,Fabp4^* and *Crif1^f/+,Fabp4^* mice. The number of *Crif1+/+* and *Crif1+/−* ADSCs was counted in 96 well plates after 10 days of differentiation (*n* = 6). Values are means + SD. n.s, not significant. (B) Oil red O staining of *Crif1+/+* and *Crif1+/−* ADSCs. (C) OXPHOS complex subunits were detected by western blotting with the appropriate antibodies. Anti-ND1 and anti-NDUFA9 were used to detect OXPHOS complex I, anti-UQCRC2 was used for complex III, and anti-COX4 was used for complex IV. (D) BN-PAGE analysis using a mixture of anti-OXPHOS antibodies to detect assembled OXPHOS complexes. (E) Oxygen consumption rates (OCR) measured by Seahorse XF-24 flux analyzer in *Crif1+/+* and *Crif1+/−* ADSCs (*n* = 6). Values are means ± SD, *p<0.05. CCCP, carbonyl cyanide m-chloro phenyl hydrazine. (F) *Crif1* mRNA expression in eWAT, BAT, liver and heart of *Crif1^+/+,Fabp4^* and *Crif1^f/+,Fabp4^* mice (*n* = 8). Values are means + SD, *p<0.05. (G) Level of ATP in eWAT of *Crif1^+/+,Fabp4^* and *Crif1^f/+,Fabp4^* mice (*n* = 8). Values are means + SD. n.s, not significant. (H) Western blots analysis of Crif1 and OXPHOS subunits from eWAT and BAT of mice. ND1 and NDUFA9, subunit of OXPHOS complex I; UQCRC2, subunit of OXPHOS complex III; COX4, subunit of OXPHOS complex IV. (I) BN-PAGE analysis of assembled OXPHOS complexes (I, II, III, IV and V). Percentage of band intensities are presented in the graph (*n* = 4). Values are means + SD, *p<0.05. (J) BN-PAGE analysis of supercomplex (I+III_2_+IV_1–4_, I+III_2_, III_2_+IV and III_2_).

A previous study revealed that *Crif1*-deficient (−/Δ) MEFs prepared from *Crif1* ^−/*flox*^ mice showed marked OXPHOS defects due to a profound failure of translation and insertion of the newly-synthesized OXPHOS polypeptides encoded by the mtDNA. Also, *Crif1* −/Δ MEFs showed increased anaerobic glycolysis, which eventually led to accelerated cell death [17]. Similar to *Crif1* −/Δ MEFs, loss of *Crif1* in adipose-derived stem cells (ADSCs) (*Crif1M−/−*) resulted in marked impairment of differentiation and accelerated cell death, which prevented functional analysis of the mitochondria (data not shown). However, control (*Crif1+/+*) and *Crif1*-haploinsufficient ADSCs (*Crif1+/−*) prepared from *Crif1^+/+,Fabp4^* and *Crif1^f/+,Fabp4^* mice showed identical levels of cell viability and differentiation to those of control cells (Figure 2A and 2B). Interestingly, *Crif1+/−* ADSCs showed lower expression of OXPHOS subunits (ND1, NDUFA9, UQCRC2 and COX4) and assembled OXPHOS complex I on Western blot and Blue Native PAGE (BN-PAGE) analysis, respectively (Figure 2C and 2D). *Crif1+/−* ADSCs consumed less oxygen under basal conditions and showed reduced maximal OXPHOS capacity (Figure 2E). Taken together, *Crif1* haploinsufficiency in adipocytes resulted in normal differentiation but reduced genetically-determined OXPHOS capacity.

Several experimental criteria have been proposed to test whether a primary *in vivo* OXPHOS deficiency plays a causal role in insulin resistance [21]. One of these criteria is that perturbations in OXPHOS gene expression and function must be as modest as possible [21]. Thus, we analyzed *Crif1* and OXPHOS gene expression to test whether *Crif1^f/+,Fabp4^* mice were suitable for our proposed experiments. Compared with *Crif1^+/+,Fabp4^* mice, *Crif1^f/+,Fabp4^* mice showed about ∼50% of the *Crif1* mRNA and protein expression in epididymal WAT (eWAT) (Figure 2F and 2H). Although basal ATP levels in eWAT were not affected by *Crif1* haploinsufficiency (Figure 2G), the expression levels of OXPHOS complex I, III and IV subunits were reduced in the epididymal fat pads of *Crif1^f/+,Fabp4^* mice (Figure 2H). BN-PAGE analysis showed that the levels of Complex I and IV and supercomplex in WAT were approximately 20%, 40% and 50% lower, respectively, in *Crif1^f/+,Fabp4^* mice compared to control mice (Figure 2I and 2J). However, normal levels of Crif1 and OXPHOS complexes were expressed in the liver and heart of *Crif1^f/+,Fabp4^* mice (Figure S3A–S3C). In contrast to homozygous *Crif1^f/f,Fabp4^* mice, heterozygous *Crif1^f/+,Fabp4^* mice exhibited normal levels of OXPHOS subunits in BAT and mitochondrial morphology was normal (Figure 2H–2J and Figure S2B).

Food intake and weight gain were comparable in *Crif1^f/+,Fabp4^* and *Crif1^+/+,Fabp4^* mice when fed a normal chow diet (NCD) (Figure S4A and S4B). MR images of control and *Crif1^f/+,Fabp4^* mice fed a NCD or a HFD showed a similar pattern of adipose distribution (Figure S4C). Triglyceride levels in the liver and plasma of *Crif1^f/+,Fabp4^* mice were the same as those in control mice, regardless of whether they were fed a NCD or a HFD. Serum free fatty acid (FFA) levels tended to be higher in *Crif1^f/+,Fabp4^* mice, but were not significantly different from those in control mice (Figure S4D–S4F). Taken together, these results show that *Crif1^f/+,Fabp4^* mice have mildly reduced primary OXPHOS deficiency in adipose tissue but, unlike the lipodystrophic model, they show no defects in adipose tissue development, and no hyperlipidemia or ectopic lipid accumulation.

### Insulin resistance in *Crif1^f/+,Fabp4^* mice

**Figure 3:**
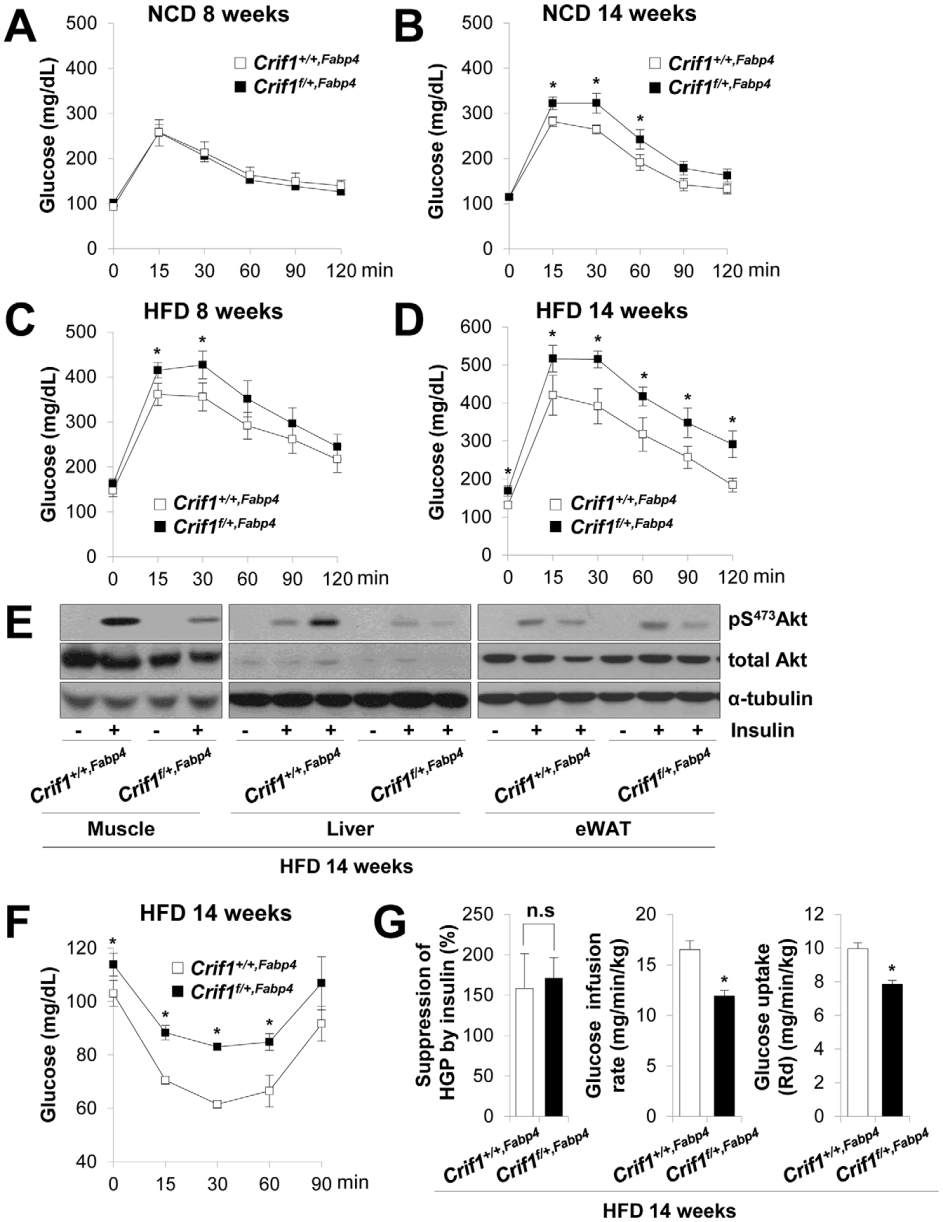
Metabolic phenotypes and insulin resistance in *Crif1^f/+,Fabp4^* mice. (A–D) An intraperitoneal glucose tolerance test (IPGTT) was performed with 1 g/kg glucose, after a 16 h fast in NCD or HFD mice (*n* = 8). Values are means ± SD, *p<0.05 versus control mice. (E) Western blot analysis of p-Akt in the gastrocnemius muscle and liver after injecting 1 U/kg insulin in control and *Crif1^f/+,Fabp4^* mice with 14 weeks of HFD. Data are representative of three independent experiments. p-Akt, phospho-Akt; t-Akt, total-Akt. (F) Insulin (0.75 U/kg) tolerance tests (ITTs) (*n* = 8). Values are means ± SD, *p<0.05 versus control mice. (G) Hyperinsulinemic euglycemic clamp analysis in *Crif1^+/+,Fabp4^* and *Crif1^f/+,Fabp4^* mice with 14 weeks of HFD. HGP, hepatic glucose production (*n* = 8). Values are means + SD, *p<0.05, n.s, not significant.

To identify the relationship between insulin resistance and reduced OXPHOS capacity in adipocytes *in vivo*, control and *Crif1^f/+,Fabp4^* mice were subjected to glucose tolerance tests after 8 weeks or 14 weeks on a NCD or HFD. Neither control nor *Crif1^f/+,Fabp4^* mice fed a NCD for 8 weeks showed any differences in glucose tolerance following an intraperitoneal injection of glucose (IPGTT, 2 g/kg body weight) (Figure 3A). However, *Crif1^f/+,Fabp4^* mice fed a NCD for 14 weeks developed glucose intolerance (Figure 3B). More impressively, *Crif1^f/+,Fabp4^* mice fed a HFD for 8 weeks showed an earlier onset of glucose intolerance, which was characterized by higher peak glucose levels than those measured in control mice in the intraperitoneal glucose tolerance tests (Figure 3C). *Crif1^f/+,Fabp4^* mice fed a HFD for 14 weeks showed more advanced glucose intolerance, with higher basal (168.8±13.2 mg/dL *vs* 131.3±8 mg/dL) and peak (516.8±34.8 mg/dL *vs* 420.4±52.3 mg/dL) plasma glucose levels (Figure 3D). Therefore, regardless of the caloric state, mice with *Crif1* haploinsufficiency showed reduced glucose tolerance. *Crif1^f/+,Fabp4^* mice fed a HFD for 14 weeks showed decreased Akt phosphorylation in the liver and muscle and a reduced glucose disposal rate after an intraperitoneal insulin challenge (Figure 3E and 3F). Furthermore, suppression of hepatic glucose production (HGP) by insulin was not different between the two groups, but the glucose infusion rate (GIR) and glucose uptake rate decreased by approximately 18.6% and 14.7%, respectively, during hyperinsulinemic euglycemic clamping after 14 weeks on a HFD (Figure 3G); these data supported the insulin tolerance tests (ITT) results. These findings indicate that *Crif1^f/+,Fabp4^* mice, which have limited OXPHOS capacity in their adipose tissue, may show exacerbated diabetic mechanisms, which are characterized by insulin resistance.

The levels of saturated fatty acids and ceramides in WAT, muscle and liver were not significantly altered in *Crif1^f/+,Fabp4^* mice (Figure S4G and S4H). Thus, abnormal accumulation of ceramides and saturated fatty acids in insulin sensitive tissues does not appear to underlie the insulin resistance of *Crif1^f/+,Fabp4^* mice (Figure S4G and S4H).

### Dysregulation of chemokines in *Crif1*-deficient differentiated adipocytes *in vitro*

**Figure 4:**
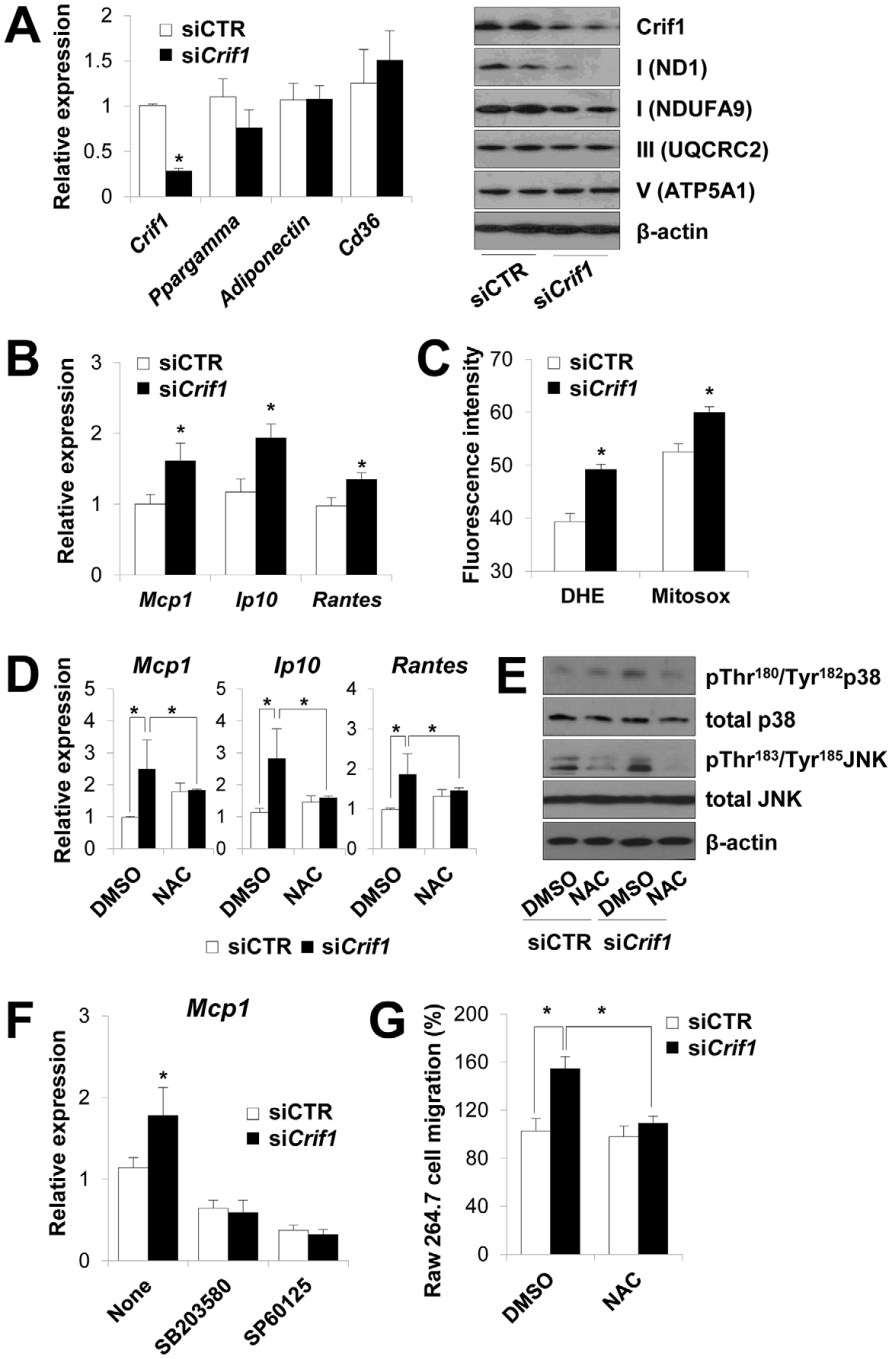
Dysregulation of chemokines and activation of stress kinases in *Crif1*-deficient 3T3-L1 adipocytes. (A) Silencing of *Crif1* and expression of adipogenic genes, *Ppargamma, adiponectin* and *Cd36* in 3T3-L1 cells. Western blot analysis with anti-ND1 and NDUFA9 (Complex I), anti-UQCRC2 (Complex III) and anti-ATP5A1 (Complex V) after 4 days of *Crif1* silencing (*n* = 10). siCTR, control siRNA; si*Crif1*, *Crif1* siRNA. Values are means + SD, *p<0.05. (B) Expression of macrophage recruiting chemokines (*Mcp1*, monocyte attractant chemokine; *Ip10*, IFN-γ–inducible protein 10; *Rantes*, Regulated upon Activation, Normal T cell Expressed and Secreted) by 3T3-L1 cells subjected to *Crif1* siRNA (*n* = 10). Values are means + SD, *p<0.05. (C) Measurement of MitoSox- or DHE-stained cells by flow cytometry. DHE, di-hydroethidium (*n* = 10). Values are means + SD, *p<0.05. (D) *Mcp1, Ip10* and *Rantes* mRNA levels with/without NAC treatment (1 mM) for 24 h. NAC, N-acetyl-L-cysteine (*n* = 10). Values are means + SD, *p<0.05. (E) p-p38 MAPK and p-JNK levels in *Crif1*-deficient 3T3-L1 adipocytes with/without NAC treatment (1 mM) for 24 h. p38 MAPK, p38 mitogen-activated protein kinases; JNK, c-Jun N-terminal kinases. (F) Inhibition of *Mcp1* expression after treatment with p-p38 (SB203580; 10 µM for 24 h) or p-JNK (SP60125; 20 µM for 24 h) inhibitors, respectively (*n* = 10). Values are means + SD, *p<0.05. (G) Migration of Raw264.7 macrophages examined using culture supernatants from control siRNA- or *Crif1* siRNA-treated 3T3-L1 adipocytes with/without NAC (*n* = 10). Values are means + SD, *p<0.05.

To determine the molecular pathways that are dysregulated by mitochondrial OXPHOS dysfunction following *Crif1* knockdown by siRNA in adipocytes, we introduced *Crif1* siRNA into fully-differentiated 3T3-L1 cells. *Crif1* knockdown in differentiated 3T3-L1 cells resulted in decreased expression of the OXPHOS subunits, ND1, NDUFA9, UQCRC2 and ATP5A1, but did not affect the expression of *Ppar-gamma, adiponectin*, and *Cd36* (Figure 4A). A complementary DNA (cDNA) microarray analysis showed prominent increases in the expression levels of inflammatory cytokine and chemokine genes in adipocytes following knockdown of *Crif1* (Figure S5). In particular, the chemokines monocyte chemotactic protein 1 (Mcp1/Ccl2), IFN-γ-inducible protein (Ip10/Cxcl10), Regulated upon Activation, Normal T cell Expressed and Secreted (Rantes/Ccl5) and Mig/Cxcl9, which are important for the recruitment of macrophages and T cells to WAT, were elevated in *Crif1*-silenced 3T3-L1 adipocytes [22]. The elevation of *Mcp1, Ip10*, and *Rantes* expression observed in cDNA microarrays was confirmed by real-time PCR experiments with *Crif1*-silenced 3T3-L1 adipocytes (Figure 4B). In parallel experiments, levels of mitochondrial and cytoplasmic superoxide anions were increased in *Crif1*-silenced 3T3-L1 adipocytes compared to control cells (Figure 4C). Treatment with the antioxidant N-acetylcysteine (NAC) suppressed the expression of *Mcp1*, *Ip10* and *Rantes* in *Crif1*-silenced 3T3-L1 adipocytes (Figure 4D).

Adipose inflammation links adipocyte dysfunction to insulin resistance, which are frequently observed in excessive adiposity (for a review, see). The inflammatory process in adipose tissue is provoked by the activation of stress kinases, e.g., c-Jun N-terminal kinase (JNK), which inhibit insulin signaling and activate transcription factors that mediate the expression of chemokine genes [24], [25]. Intracellular stress signals including mitochondrial ROS, FFA, ceramide and ER stress activates the stress kinases, JNK, p38 MAPK and NF-κB in adipocytes [4], [26], [27]. JNK mediates macrophage activation and expression of proinflammatory cytokines and inhibits insulin receptor substrate 1 (IRS-1)-mediated insulin signaling pathways (for a review, see [28], [29]). To identify the roles of stress kinases in the expression of chemokines in *Crif1*-silenced 3T3-L1 adipocytes, p38 MAPK and JNK phosphorylation were observed by Western blot analysis. Levels of phosphorylated p38 MAPK and JNK were elevated in *Crif1*-silenced 3T3-L1 adipocytes compared to control cells; however, this activation was suppressed by NAC treatment (Figure 4E). These results indicate that chemokine dysregulation is associated with increased ROS generation and inappropriate activation of p38 MAPK and JNK. To confirm these results, 3T3-L1 cells were treated with inhibitors of p38 MAPK and JNK (SB203580 and SP60125, respectively). Two inhibitors effectively inhibited the expression of *Mcp1* in *Crif1* silenced 3T3-L1 cells (Figure 4F). *Crif1* deficiency in MEFs results in increased ROS production [17] and induces phosphorylation of p38 (Figure S6A). However, *Crif1* -/Δ MEFs did not show increased *Mcp1* and *Ip10* expression (Figure S6B). Taken together, these results suggest that limited mitochondrial OXPHOS function in fully-differentiated adipocytes triggers the expression of chemokines (*Mcp1*, *Ip10* and *Rantes*) in a cell-specific manner.

The chemokines (*Mcp1*, *Ip10* and *Rantes*) upregulated in *Crif1* siRNA-treated adipocytes are thought to be critical for attracting macrophages and T lymphocytes into adipose tissue in obese subjects [30]. Therefore, we wondered whether *Crif1*-silenced 3T3-L1 cells would trigger the migration of macrophages. As shown in Figure 4G, *Crif1*-silenced 3T3-L1 cells enhanced the migration of RAW 264.7 cells and NAC treatment inhibited the migration of RAW 264.7 cells. Thus, our *in vitro* studies show that OXPHOS deficiency induced in differentiated cultured 3T3-L1 adipocytes by *Crif1* silencing results in the upregulated expression of chemokines, which then recruit or activate macrophages, ROS and stress kinase dependently.

### Macrophage infiltration and systemic inflammatory responses in *Crif1^f/+,Fabp4^* mice

**Figure 5:**
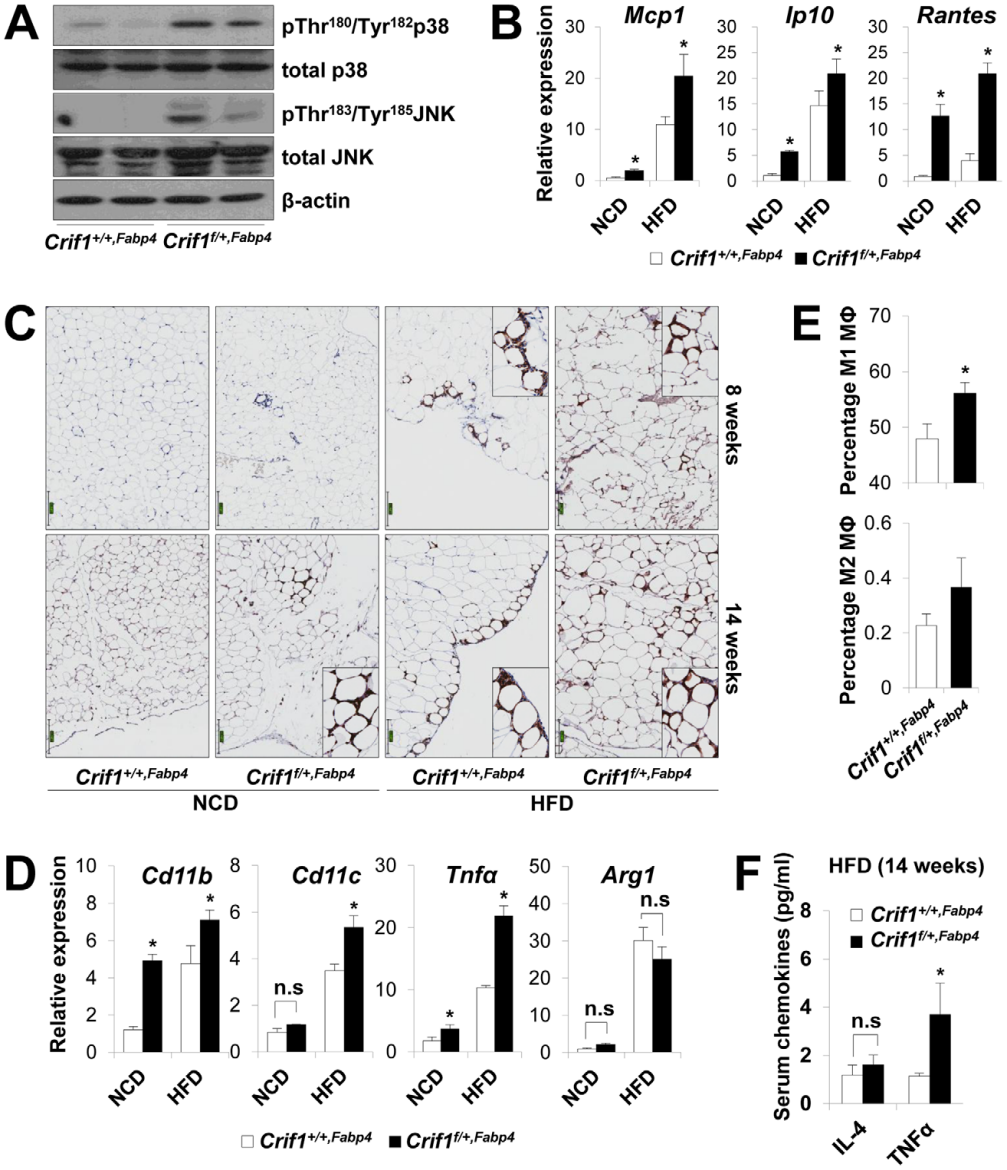
Macrophage infiltration and inflammation in adipose tissue of *Crif1^f/+,Fabp4^* mice. (A) Western blotting for p38 MAPK and JNK phosphorylation in WAT of *Crif1^f/+,Fabp4^* mice fed a HFD for 8 weeks. p38 MAPK, p38 mitogen-activated protein kinase; JNK, c-Jun N-terminal kinase. (B) *Mcp1*, *Ip10* and *Rantes* mRNA levels in eWAT from control mice fed a NCD or a HFD for 8 weeks (*n* = 8). Values are means + SD, *p<0.05 versus control mice. (C) Immunohistochemistry with anti-F4/80 and hematoxylin staining reveals macrophage accumulation in eWAT. Scale: 200 µm. (D) Expression of the M1 macrophage and M2 macrophage-specific genes (*Cd11c, Cd11b, Tnfα* and *arginase1*) in eWAT (*n* = 8). Values are means + SD, *p<0.05 versus control mice, n.s, not significant. (E) Percentage of proinflammatory M1 macrophages and anti-inflammatory M2 macrophages assessed by counting CD11c-PE-A- and CD206-FITC-stained cells in the SVF in eWAT of 14 weeks HFD feeding mice (*n* = 8). Values are means + SD, *p<0.05 versus control mice. MΦ, Macrophage. (F) Serum IL4 and TNFα levels in mice fed HFD, as measured by Multiplex (*n* = 8). Values are means + SD, *p<0.05 versus control mice, n.s, not significant.

To observe ROS stress associated with abnormal chemokine responses in adipose tissues *in vivo*, we measured lipid peroxidation (TBAR assays), stress kinase activation and cytokine expression in WAT of *Crif1^f/+,Fabp4^* mice fed a NCD or a HFD for 8 weeks. Consistent with the *in vitro* studies, lipid peroxidation in WAT and plasma was increased in *Crif1^f/+,Fabp4^* mice fed a HFD for 8 weeks compared to control mice (Figure S7A). Levels of p38 MAPK and JNK phosphorylation were higher in WAT of *Crif1^f/+,Fabp4^* mice fed a HFD for 8 weeks than in control mice (Figure 5A). Furthermore, the expression of *Mcp1*, *Ip10* and *Rantes* was higher in adipose tissue from *Crif1^f/+,Fabp4^* mice than in control mice (Figure 5B). In addition, the level of secreted MCP1, but not IP10, were higher in the serum of *Crif1^f/+,Fabp4^* mice than in control mice (Figure S7B). The results showing dysregulation of chemokines in the absence of *Crif1* suggest that mitochondrial OXPHOS dysfunction may trigger immune cell recruitment in adipose tissue.

To observe inflammation in the adipose tissue of *Crif1^f/+,Fabp4^* mice directly, the eWAT were stained with anti-F4/80, an antibody that detects macrophages. Increased F4/80 reactivity was observed in the eWAT of *Crif1^f/+,Fabp4^* mice fed a NCD for 8 weeks. Aging and a HFD had an even more pronounced effect (Figure 5C). Based on the quantitative real-time PCR results, the relative expression of proinflammatory M1 macrophage markers (*Cd11c, Cd11b* and *Tnfα*) increased significantly; however, the relative gene expression of an anti-inflammatory M2 macrophage marker (*arginase 1*) did not change (Figure 5D). To quantify the number of macrophages in the adipose tissue, multi-parameter flow cytometry was performed with anti-F4/80, anti-CD11c and anti-CD206 antibodies using isolated stromal vascular fractions (SVF). F4/80+/CD11c+/CD206- M1 macrophages were predominant in *Crif1^f/+,Fabp4^* mice compared with control mice. The proportion of F4/80+/CD11c-/CD206+ M2 macrophages tended to be higher in *Crif1^f/+,Fabp4^* mice, but this did not reach statistical significance (Figure 5E). Taken together, the results suggested that the infiltrating macrophages were skewed towards the M1 phenotype in *Crif1^f/+,Fabp4^* mice. Recent studies show that B cell-mediated CD4^+^ and CD8^+^ T cell activation is required to induce inflammation and insulin resistance [31], [32]. The present study found no difference between the numbers of CD4^+^ and CD8^+^ T cells in *Crif1^+/+,Fabp4^* and *Crif1^f/+,Fabp4^* mice (data not shown).

Adipocytes in adipose tissue secretes adipokines, such as adiponectin, leptin, IL-6 and TNFα, which are involved in the control of whole-body insulin sensitivity. However, proinflammatory TNFα is released by dysfunctional adipocytes and amplifies local immune responses by recruiting macrophages to WAT [33], [34]. Serum levels of TNFα were consistently higher in *Crif1^f/+,Fabp4^* mice fed a HFD than in control mice (Figure 5F). This indicates that TNFα may be a crucial mediator of inflammation in WAT and whole-body insulin resistance of *Crif1^f/+,Fabp4^* mice.

The Fabp4 gene is expressed in macrophages [35], but no *Cre* expression or activity was detected in macrophages isolated from *Crif1^f/+,Fabp4^* mice. As shown in Figure S8A, the expression levels of *Crif1* and macrophage markers (*Cd11c, Tnfα, Cd11b*, and *Arg1*) were not reduced in peritoneal macrophages obtained from *Crif1^f/+,Fabp4^* mice (Figure S8A). Homologous recombination using PCR [36] identified *Cre* recombinase activity in WAT and BAT, but not in peritoneal macrophages in *Crif1^f/+,Fabp4^* mice at 20 weeks-of-age (Figure S8B and S8C).

### Adipose inflammation and glucose intolerance in *Crif1^f/f,Adipoq^* mice

To verify the adipose inflammation characterized by macrophage infiltration in *Crif1*-null mice, we generated another adipose tissue-specific *Crif1* knockout mouse by crossing floxed *Crif1* mice with Adipoq-*Cre* transgenic mice. Adipoq-*Cre* transgenic mice expressed *Cre* recombinase in WAT and BAT, but not in macrophages (including adipose tissue resident macrophages, alveolar macrophages, or thioglycolate-stimulated peritoneal macrophages) [37].

The homozygous *Crif1* knockout mice (*Crif1^f/f,Adipoq^*) showed about ∼30% of the *Crif1* expression observed in the eWAT of controls (Figure S9A). They showed decreased expression of OXPHOS subunits (ND1, NDUFA9, UQCRC2 and COX4) in eWAT and BAT, not in liver and heart (Figure S9B). We compared adipocyte development in the adipocyte-specific *Crif1* knockout mouse with that of Adipoq-*Cre* mice. H&E staining of adipose tissues indicated that the adipocytes of *Crif1^f/f,Adipoq^* mice were relatively smaller and irregularly shaped in comparison to those of *Crif1^+/+,Adipoq^* mice (data not shown).

**Figure 6:**
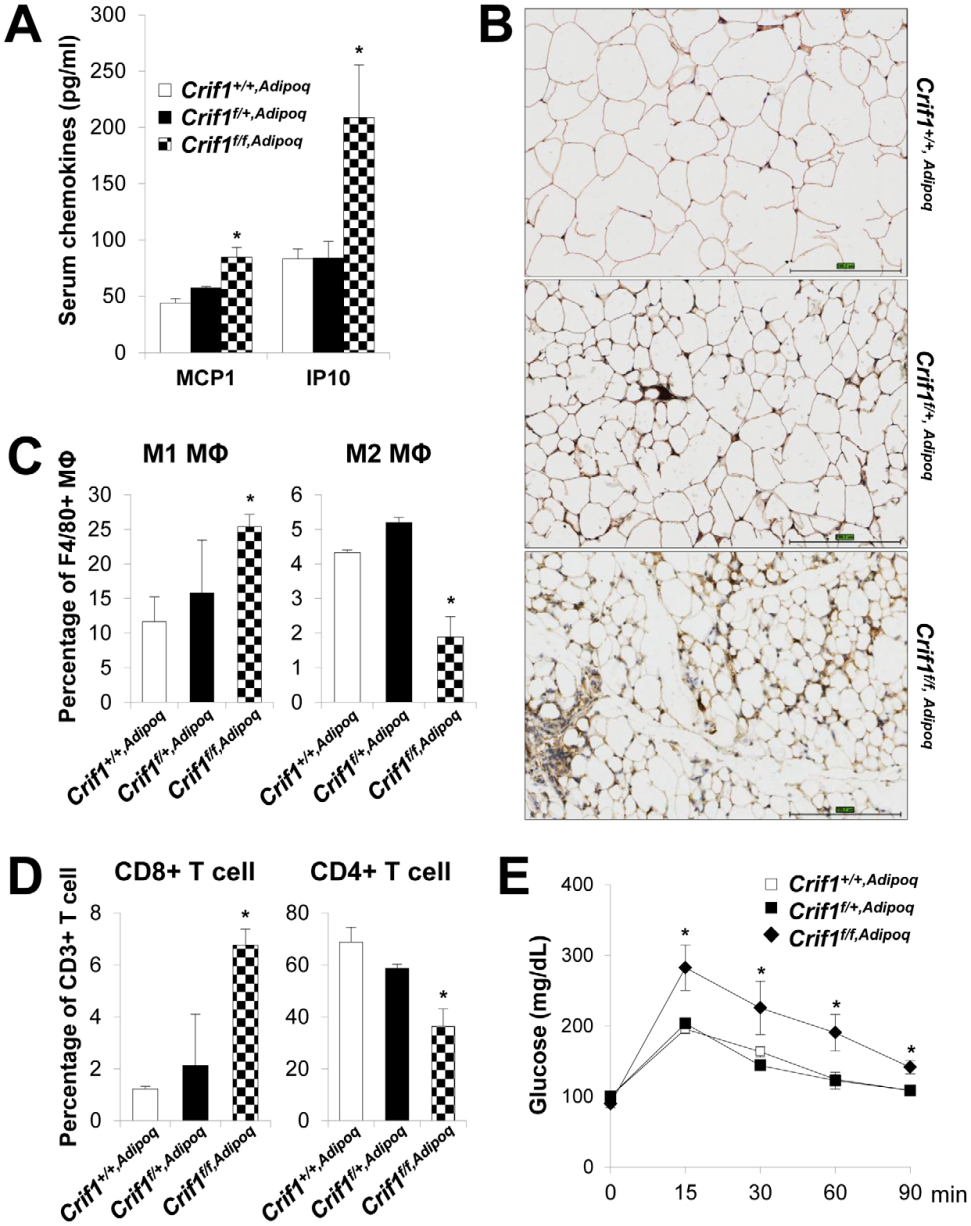
Recruitment of macrophages and T cells to adipose tissue in *Crif1^f/f,Adipoq^* mice. Control (*Crif1^+/+,Adipoq^*), adipose-specific *Crif1* heterozygous (*Crif1^f/+,Adipoq^*), and homozygous (*Crif1^f/f,Adipoq^*) knockout mice were generated by cross-breeding with Adipoq-*Cre* recombinase transgenic mice. Phenotypic analysis all of these mice fed with a NCD was performed at 8 weeks-of-age. (A) Secreted MCP1 and IP10 levels in the serum in the three strains of mice (*n* = 6). Values are means + SD, *p<0.05 versus control mice. (B) Immunohistochemical staining with an anti-F4/80 antibody shows severe macrophage accumulation in eWAT of *Crif1^f/f,Adipoq^* mice. Scale: 200 µm. (C) Percentage of proinflammatory M1 macrophages (CD11c-PE-A-) and anti-inflammatory M2 macrophages (CD206-FITC-) (*n* = 6). Values are means + SD, *p<0.05. MΦ, Macrophage. (D) Percentage of CD8 positive T cells and CD4 positive T cells in the SVF in eWAT. Quantification of the CD8/CD4 ratio by counting cells stained with CD8-FITC and CD-PE-cy7 (*n* = 6). Values are means + SD, *p<0.05. (E) IPGTT was performed in NCD mice after a 16 h fast with 1 g/kg of glucose (*n* = 6). Values are means ± SD, *p<0.05 versus control.

Consistent with the *Crif1^f/+,Fabp4^* mouse model, *Crif1^f/f,Adipoq^* mice showed higher plasma levels of MCP1 (1.9-fold higher), IP10 (2.5-fold higher) and marked F4/80 immuno-reactivities in eWAT, suggesting inflammation in WAT (Figure 6A and 6B). The nature of the macrophage phenotypes was further identified by flow cytometry using fluorescently-labeled anti-F4/80, anti-CD11c, and anti-CD206 antibodies. In addition, the T cell population was also analyzed using anti-CD3, anti-CD8, and anti-CD4 antibodies. *Crif1^f/f,Adipoq^* mice had a higher level of M1 macrophages and a lower level of M2 macrophages in eWAT compared to *Crif1^+/+,Adipoq^* mice (Figure 6C). The level of cytotoxic CD8-positive T cells was increased 5.5-fold and the level of CD4-positive helper T cells was decreased 0.5-fold in *Crif1^f/f,Adipoq^* in comparison to control mice; however, the levels of these cells in *Crif1^f/+,Fabp4^* mice were not significantly different from control mice (Figure 6D). This reflect differences in the severity of the defect in the mitochondrial OXPHOS complex in *Crif1^f/f,Adipoq^* and *Crif1^f/+,Fabp4^*mice. Similar to *Crif1^f/+,Fabp4^* mice, *Crif1^f/f,Adipoq^* mice developed glucose intolerance even in being fed a NCD, at 8 weeks-of-age (Figure 6E).

Unlike *Crif1^f/f,Fabp4^* mice, *Crif1^f/f,Adipoq^* mice were viable. This discrepancy could be due to the inherent differences in the activities of *Cre*-recombinase driven by Fabp4 and adiponectin promoter (Table S1). Loss of Crif1 was consistently observed in WAT in both mouse lines; however, the degree of Crif1 loss was more severe in *Crif1^f/f,Fabp4^* mice than in *Crif1^f/f,Adipoq^* mice. Also, *Crif1^f/f,Fabp4^* mice exhibited a severe loss of BAT and WAT mass, whereas the mass of these tissues was only mildly reduced in *Crif1^f/f,Adipoq^* mice. Consistent with these findings, homozygous *Crif1^f/f,Fabp4^* mice rapidly reached a fatal low rectal temperature of 22.6+1.9°C (Figure S2D), whereas homozygous *Crif1^f/f,Adipoq^* mice reached a milder rectal temperature of 29.5+0.7°C within 5 minutes of immersion in cold water (4°C) (Figure S9C). These results indicated that thermal stress caused by mitochondrial OXPHOS dysfunction in BAT following *Crif1* ablation may be a causative factor of the premature death of adipocyte specific *Crif1* knockout mice, and that BAT dysfunction may be partially involved in systemic glucose intolerance.

### Reversal of insulin resistance in adipose-specific *Crif1*-deficient mice by clodronate treatment

**Figure 7:**
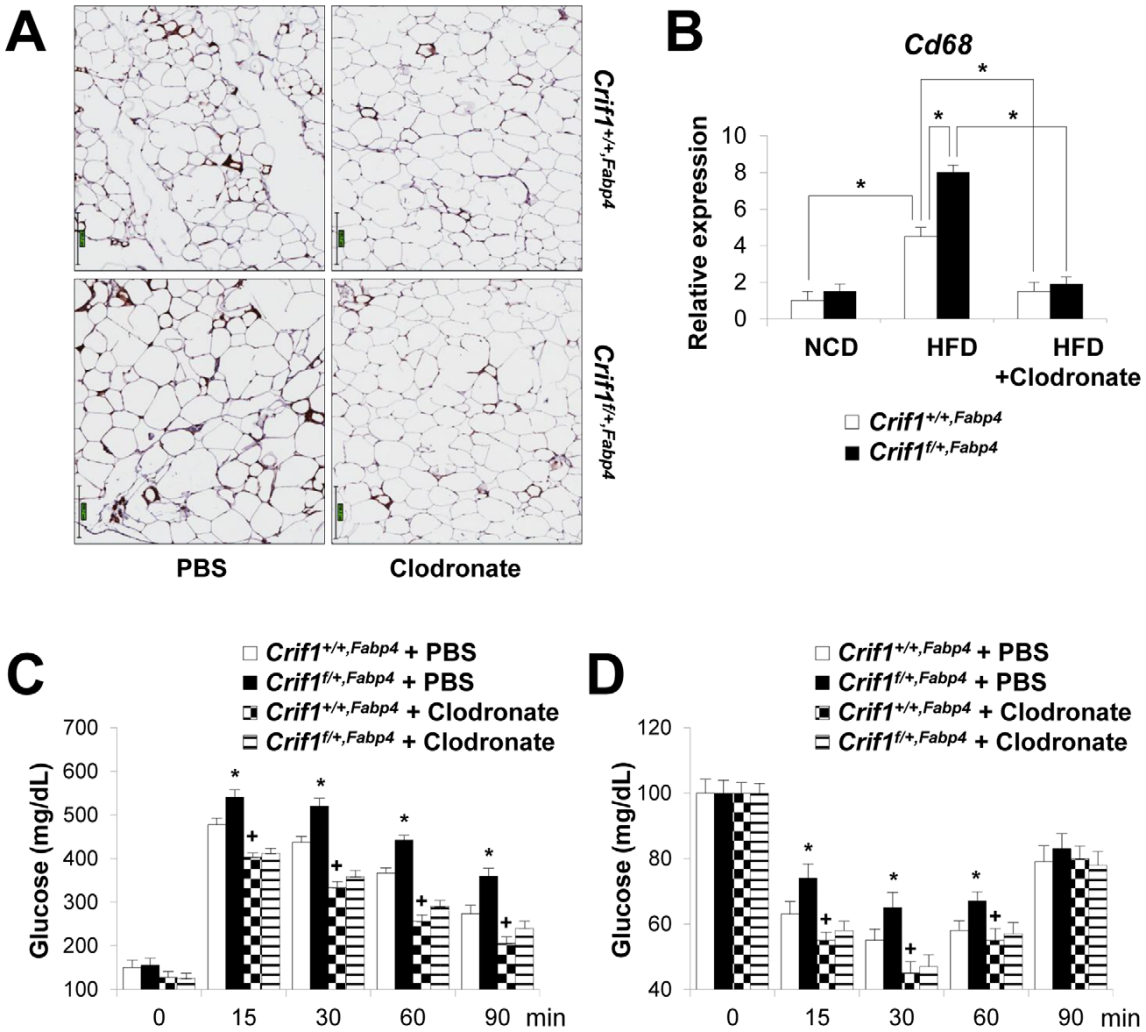
Depletion of macrophages in adipose tissue by clodronate. The clodronate study was performed after feeding *Crif1^+/+,Fabp4^* and *Crif1^f/+,Fabp4^* mice a HFD for 8 weeks. Two intraperitoneal injections of clodronate were given with a 3 day interval between each. IPGTT and ITT were performed 6 days after the first injection. (A) Immunohistochemistry with anti-F4/80 after macrophage depletion by liposomal clodronate in *Crif1^+/+,Fabp4^* and *Crif1^f/+,Fabp4^* mice fed HFD. Scale: 200 µm. (B) Real-time PCR using primers for the macrophage marker, *Cd68* (*n* = 8). Values are means + SD, *p<0.05. (C and D) IPGTT and ITT after macrophage depletion by intraperitoneal injection of liposomal clodronate or PBS control (*n* = 8). Values are means + SD, *p<0.05, *Crif1^f/+,Fabp4^* mice versus control mice; + p<0.05, clodronate versus PBS in control mice.

**Figure 8:**
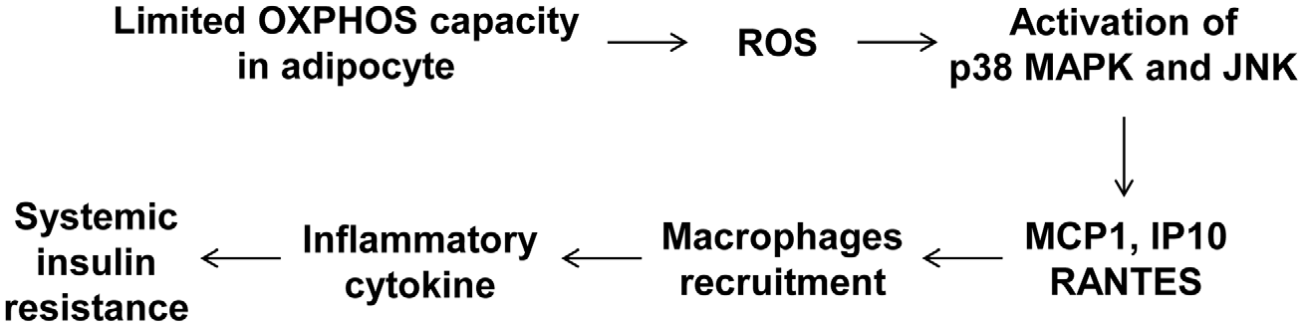
A model of the systemic insulin resistance developed by *Crif1*-haploinsufficient mice, which shows limited adipose OXPHOS capacity.

To determine whether macrophages in the adipose tissue of *Crif1^f/+,Fabp4^* mice play a role in insulin resistance, we depleted macrophages from adipose tissue by intraperitoneal treatment with clodronate liposomes [38]. Clodronate is an apoptosis-inducing drug; therefore, injection of liposome-encapsulated clodronate into the intraperitoneal cavity can deplete phagocytic cells, such as macrophages. Control and *Crif1^f/+,Fabp4^* mice fed a HFD for 8 weeks were intraperitoneally administered two rounds of clodronate liposomes with an interval of 3 days. The accumulation of macrophages positively stained with an anti-F4/80 antibody was decreased in the eWAT following injection of clodronate liposomes (Figure 7A). Furthermore, the level of *Cd68* mRNA was significantly lower in the eWAT of mice injected with clodronate than in untreated mice (Figure 7B). Administration of clodronate liposomes dramatically improved the insulin and glucose tolerance of *Crif1^f/+,Fabp4^* mice fed a HFD for 10 weeks (Figure 7C and 7D). These findings indicate that suboptimal reserves of mitochondrial OXPHOS in the adipose tissue of *Crif1*-deficient mice induce macrophage recruitment, which may trigger systemic insulin resistance (Figure 8).

## Discussion

Mitochondrial dysfunction, characterized by reduced OXPHOS function in liver and skeletal muscle, is thought to be one of the underlying causes of insulin resistance and type 2 diabetes (for a review, see [39], [40]). In addition, reduced hepatic OXPHOS function is closely related to hepatic lipid accumulation and insulin resistance [41]. Collectively, these studies provide evidence of a role for mitochondrial OXPHOS dysfunction in the development of human insulin resistance and type 2 diabetes. However, animal models of OXPHOS dysfunction in skeletal muscle and liver do not exhibit the human insulin resistance and type 2 diabetes phenotype [21], [42], [43]. The absence of insulin resistance in mice with homozygous or heterozygous *Crif1* deletion in the liver (*Crif1^f/f,Alb^*, Albumin-*Cre*) or skeletal muscle (*Crif1^f/+,MLC^*, MLC-*Cre*) is in agreement with previous findings that hepatic and skeletal mitochondrial dysfunction does not cause insulin resistance (Figure S10). Therefore, whether or how mitochondrial OXPHOS contributes to the pathogenesis of insulin resistance remains to be resolved. It is reported that adipose OXPHOS capacity is controlled by both genetic and diet-induced obesity [10], [44], [45], which potentially contribute to adipose tissue dysfunction and exacerbation of insulin resistance. However, whether changes in adipose OXPHOS capacity are a cause or a consequence of complications associated with insulin resistance has not been clarified *in vivo*.

In this study, we have shown an association between limited mitochondrial OXPHOS capacity and adipose tissue inflammation and insulin resistance in a *Crif1* haploinsufficiency animal model. Mitochondria play a key role in the differentiation and maturation of adipocytes. It is reported that marked mitochondrial biogenesis is observed during the adipocyte differentiation process *in vitro*. In fact, the concentration of mitochondrial proteins in differentiated 3T3-L1 adipocytes showed a 20- or 30-fold increase compared with that in pre-adipocytes [14], [46]. Notably, chemical inhibition of respiratory chain function, for example by rotenone treatment, suppresses adipogenesis and induces changes in the expression levels of the key transcription factors, C/EBPα, PPARγ, and SREBP-1c [47]. However, the role played by mitochondria during adipogenesis has mostly been investigated *in vitro* by inhibiting or knocking down the genes encoding the OXPHOS complex. This study showed that homozygous *Crif1* null mice generated by both Fabp4-*Cre* and Adipoq-*Cre* recombinase have defects in WAT and BAT development. These observations support that notion that intact OXPHOS function is critical for adipogenesis *in vivo*. By contrast, our own observations show that heterozygous *Crif1* knockout mice do not have defects in adipogenesis and maturation under NCD and HFD conditions. This finding indicates that *Crif1* haploinsufficiency and mildly reduced OXPHOS capacity do not cause the apparent failure of adipogenesis in WAT and BAT. Consistently, plasma and liver lipid levels were not increased in heterozygous *Crif1* knockout mice, suggesting that the mice do not have the lipodystrophy phenotype. Furthermore, it is reported that insulin resistance in a mouse model of lipodystrophy was not relieved by controlling inflammation [48]. Therefore, insulin resistance in *Crif1* haploinsufficient knockout mice may not be related to lipodystrophic changes.

Similar to WAT, the development of BAT was severely perturbed in homozygous *Crif1*-null mice (*Crif1^f/f,Fabp4^*), which may be a critical factor in the early mortality of these mice. By contrast, BAT development was normal in heterozygous *Crif1^f/+,Fabp4^* mice, and the histology and ultrastructure of mitochondria were normal. Furthermore, core temperature responses to a cold environment suggest that Fabp4-*Cre*-driven haploinsufficiency of *Crif1* may not affect the physiological function of BAT. Therefore, decreased BAT function and impaired energy expenditure may not be principal cause of the development of insulin resistance in *Crif1*-haploinsufficient mice.

The OXPHOS capacity in adipose tissue may be controlled by tissue-specific, genetic and environmental factors. In fact, it is well known that each cell type develops and maintains a specific OXPHOS capacity to satisfy its metabolic and energetic demands. In addition, individual OXPHOS capacity is genetically determined by specific tissues [49]. The cellular and genetic factors that control adipose-specific OXPHOS capacity are not fully understood. Therefore, white adipocyte responses to marginal or limited OXPHOS capacity *in vitro* and *in vivo* remain to be elucidated. In the present study, we characterized the enhanced secretory chemokine responses in *Crif1*-silenced mature adipocytes. Chemokine production in WAT is physiological, but enhanced production is linked to adipose inflammation, which is usually observed in cases of excessive adiposity (for a review, see [50]). Therefore, the earliest events that trigger the process of enhanced chemokine secretion are of great interest. Studies on signals that initiate adipose inflammation are mainly based on the model of ER homeostasis, lipolysis, and fatty acid signals in obese individuals (for a review, see [4]). It is not known how mitochondria or OXPHOS dysfunction modify the chemokine responses in WAT under physiological and pathological conditions. We found that abnormal increases in ROS production and activation of p38 and JNK were associated with increased expression of *Mcp1*, *Ip10* and *Rantes*. The increase in ROS and the activation of p38 and JNK in adipose tissue are common denominators that respond to cellular stresses. Unexpectedly, haploinsufficient heterozygous *Crif1^f/+,Fabp4^* mice and control mice exhibited similar levels of adipose Akt phosphorylation in response to insulin injection. This indicates that insulin signaling in adipose tissue may not be the principal cause of the systemic glucose intolerance of *Crif1^f/+,Fabp4^* mice. Therefore, the relative importance of these factors (increased ROS, p38 and JNK activation) need to be addressed by suppressing or eliminating these events while studying abnormal chemokine responses and systemic insulin resistance.

We showed that the WAT in *Crif1*-deficient mice is predominantly infiltrated by macrophages, regardless of excessive adiposity. An increase in the number of adipose tissue macrophages (ATM) is a prominent feature associated with excessive adiposity [51], [52]. Increased expression of chemokines, especially MCP1, is responsible for recruiting macrophages into the WAT [30]. The ATM infiltration of epididymal fat pads in *Crif1*-deficient mice showed several characteristic features. First, it was present in fat pads with normal adiposity. This finding suggests that macrophage recruitment to adipose tissue caused by impaired OXPHOS capacity may also develop independently of excessive adiposity, but is accentuated in cases of increased adiposity. Mitochondrial OXPHOS dysfunction in the adipose tissue of *Crif1^f/+,Fabp4^* mice fed a NCD for 8 weeks resulted in macrophage recruitment; however, the mice showed normal glucose tolerance. This suggests that a threshold level of macrophage recruitment or activation is required for the development of insulin resistance. Phenotypic analysis of ATM in *Crif1*-deficient mice demonstrated that the proportion of both M1 and M2 macrophages tended to be increased under NCD and HFD conditions. However, a phenotypic shift toward M1 macrophages was observed in the adipose tissue of *Crif1*-deficient mice. Thus, these features of macrophage recruitment in WAT were similar to those observed in a mouse model of diet-induced obesity [53].

Our data provide novel insights into the relationship between adipose inflammation and insulin resistance. This study supports the idea that adiposity overwhelms the genetically-determined OXPHOS capacity in adipose tissue, provoking an inflammatory response and insulin resistance. Therefore, it is possible that adipose mitochondrial OXPHOS capacity is an independent factor determining the risk of adipose inflammation and systemic insulin resistance in obese and even in non-obese subjects.

## Materials and Methods

### Cell culture

3T3-L1 cells were maintained in Dulbecco's modified Eagle's medium (DMEM) supplemented with 10% bovine calf serum (Gibco BRL). Forty-eight hours post-confluence, the cells were differentiated with IBMX (0.5 mM), dexamethasone (1 µM), insulin (10 µg/ml) and 10% fetal bovine serum (Gibco BRL) [54]. *Crif1* siRNA (GGA GUG CUC GCU UCC AGG AAC UAU U) was transfected by using Lipofectamine RNAiMAX reagent (Invitrogen) into 3T3-L1 adipocytes on day 4 of differentiation. Migration of Raw264.7 cells was examined in 8.0 µm Transwell filters (Corning Corp). Raw 264.7 cells were maintained on the top well, with the media from 3T3-L1 adipocytes in the bottom well. After twenty-four hours, the Raw 264.7 cells that had not migrated to the filter were removed, and the cells that had migrated through the filter were collected and stained with trypan-blue. ADSCs were cultured as previously described [55]. ADSCs were differentiated into adipocytes using IBMX (0.5 mM), dexamethasone (1 µM), insulin (10 µg/ml) and rosiglitazone (0.5 µM) in M199 medium (Gibco BRL) supplemented with 10% fetal bovine serum (Gibco BRL). After induction of differentiation, lipid accumulation was detected with Oil red O staining. ADSCs were fixed with 10% neutralized formalin, washed with water, and then stained with freshly prepared 0.2% Oil red O solution.

### Western blot analysis

Primary antibodies against OXPHOS complex subunits (NDUFA9, SDHA, UQCRC2, and ATP5A1) were purchased from Invitrogen. Anti-COX4 (#4844) antibody was purchased from Cell Signaling. Anti-ND1 antibody (sc-65237) was purchased from Santa Cruz Biotechnology. Secondary antibodies (goat anti-mouse and goat anti-rabbit) were obtained from Cell Signaling. Anti-p38 antibody, anti-phospho-p38 antibody, anti-JNK-antibody, anti-phospho-JNK antibody, anti-phospho-Akt and total-Akt antibodies were obtained from Cell Signaling and anti-β-actin, α-tubulin antibody was obtained from Sigma-Aldrich. Anti-UCP1 antibody was obtained from Abcam.

### Northern blot analysis and real-time PCR

Total RNA was isolated using Trizol (Invitrogen). For Northern blot analysis, 10–20 µg of total RNA was loaded onto a 1.5% agarose-formaldehyde gel. A *Crif1* probe was constructed using the mouse *Crif1* gene digested with KpnI enzyme. The relative intensity of the Crif1/β-actin bands was normalized against that in the brain. Complementary DNA (cDNA) was prepared from total RNA using M-MLV Reverse Transcriptase and oligo-dT primers (Invitrogen). Real-time PCR was performed using cDNA, QuantiTect SYBR Green PCR Master Mix (QIAGEN), and specific primers. The primers used are described in Table S2. Relative expressions were calculated normalized with 18s ribosomal RNA, using Rotor-Gene 6000 real-time rotary analyzer Software (Version 1.7, Corbett Life Science).

### Complementary DNA microarray analysis

Total RNA was prepared from fully-differentiated 3T3-L1 adipocytes transfected with control or *Crif1* siRNA. RNA amplification and labeling were performed with the Low RNA Input Linear Amplification kit PLUS (Agilent Technologies). Array hybridization and scanning were performed with a DNA microarray Chip and scanner (Agilent Technologies). Array data was analyzed using the Feature Extraction and GeneSpring Software (Agilent Technologies).

### ROS staining

Dihydroethidium (DHE) or MitoSOX were used to detect intracellular superoxide. Fully-differentiated 3T3-L1 cells were incubated with 10 µM DHE or 5 µM MitoSOX at 37°C for 15 min. Fully-differentiated 3T3-L1 cells were washed with Krebs-HEPES buffer (pH 7.4) or HBSS. Images of cells stained with DHE or MitoSOX were obtained by fluorescence microscopy (Olympus, Japan). Cells were trypsinized and analyzed using a FACScan flow cytometer (BD Bioscience) and data analysis was performed using BD FACSDiva software (BD Bioscience).

### Blue native-polyacrylamide gel electrophoresis (BN-PAGE)

Before BN-PAGE, mitochondrial isolation was performed as previously described [56] with modifications. Pellets of ADSCs or tissues from mice were resuspended in buffer B (210 mM mannitol, 70 mM sucrose, 1 mM EGTA, and 5 mM HEPES, pH 7.2) and incubated for 5 min at 4°C. After centrifugation at 600× g for 10 min, the supernatant was re-centrifuged at 17,000× g for 10 min. The pellet containing the mitochondrial fraction was used in the Native PAGE Novex Bis-Tris Gel system (Invitrogen) to determine the content of the OXPHOS complex. A total of 20 µg of the mitochondrial fraction in Native PAGE sample buffer supplemented with 0.5% n-dodecyl-β-D-maltoside was loaded onto a Native PAGE Novex 3–12% Bis-Tris gel. The mitochondrial fraction was mixed with Native PAGE sample buffer containing 1% of digitonin to detect the supercomplexes. After electrophoresis, the separated proteins in the gel were transferred to a PVDF membrane, which was then incubated with an anti-OXPHOS antibody mixture (Invitrogen).

### Oxygen consumption rate (OCR)

OCR was measured using a Seahorse XF-24 analyzer (Seahorse Bioscience). Control *Crif1+/+* and *Crif1+/−* ADSCs were prepared from the eWAT of *Crif1^+/+,Fabp4^* and *Crif1^f/+,Fabp4^* mice. After seeding ADSCs on an XF-24 plate, cells were incubated in differentiation M199 media contained with FBS, IBMX, dexamethasone, insulin and rosiglitazone. After 2 days later, *Crif1+/+* and *Crif1+/−* ADSCs maintained M199 media with insulin for 8 days. The day before OCR measurement, the sensor cartridge was calibrated with calibration buffer (Seahorse Bioscience) at 37°C. Fully-differentiated ADSCs were washed and incubated with M199 (Gibco BRL) without sodium bicarbonate at 37°C in an incubator. Three readings were taken after each addition of mitochondrial inhibitor before injection of the subsequent inhibitors. The mitochondrial inhibitors used were oligomycin (2 µg/ml), carbonyl cyanide m-chloro phenyl hydrazine (CCCP, 10 µM), and rotenone (1 µM). OCR was automatically calculated and recorded by the sensor cartridge and Seahorse XF-24 software. The plates were saved and the protein concentration was calculated to confirm that there were an approximately equal number of cells in each well.

### Mice

Floxed *Crif1* (*Crif1^flox/flox^*) mice were generated as previously described [18]. Fabp4-*Cre*, Albumin-*Cre* transgenic mice (C57BL/6J) were purchased from the Jackson Laboratory. Adiponectin-*Cre* transgenic mice were kindly provided by Dr. Evan Rosen. Dr. Steven J Burden provided the MLC-*Cre* mouse strain. The HFD, which contained 60% fat, was purchased from Research Diets Inc. (D12492). Mice were maintained in a controlled environment (12 h light/12 h dark cycle; humidity 50–60%; ambient temperature 23°C±1°C) and fed ad libitum. For the cold challenge experiments, mice were individually housed in cages pre-chilled to 4°C. Body temperature was monitored using a rectal probe attached to a digital thermometer (TD-300, Shibaura Denshi. Japan) with/without cold stress. For the thermoneutrality experiments, 2-week-old mice were housed with their mothers at a temperature of 30°C±1°C. All mouse experiments were performed in the animal facility according to institutional guidelines, and the experimental protocols were approved by the institutional review board of Korean Research Institute of Biotechnology and Bioscience, and Chungnam National University.

### Genomic PCR

To measure the activity of *Cre* recombinase, PCR was performed as previously reported [36]. Briefly, after isolation of genomic DNA from WAT, BAT, and thioglycolate-induced peritoneal macrophages, PCR was performed with a combination of three primers: forward primer 1, GGGCTGGTGA AATGTGTTG; reverse primer 2, TCAGCTAGGG TGGGACAGA; and reverse primer 3, TATCAGTCCG AGAAGACCTG. To ensure product specificity from PCR, the extension time was limited to 30 sec.

### Histological and morphometric analysis

WAT was fixed in 10% neutralized formalin for 16 h, washed, and then embedded in paraffin. Tissue sections of 5 µm thickness were deparaffinized, rehydrated, and heated in a microwave for 10 min in citrate buffer. The tissue sections were then incubated with primary antibodies (anti-F4/80 (diluted 1∶100; Abcam)) for 16 h at 4°C. Immunohistochemistry was performed using a Polink-1 HRP Rat-NM DAB Detection System (GBI Inc).

### Transmission electron microscopy (TEM)

WAT and BAT were fixed in 1% glutaraldehyde at 4°C and then washed with 0.1 M cacodylate buffer at 4°C. After washing five times, the tissue was post-fixed with 1% OsO_4_ in an 0.1 M cacodylate buffer (pH 7.2) containing 0.1% CaCl_2_ for 1 h at 4°C. Samples were dehydrated by serial ethanol and propylene oxide treatment and embedded in Embed-812 (EMS). The resin was then polymerized at 60°C for 36 h. Tissue was sectioned using an EM UC6 ultramicrotome (LEICA) and stained with 4% uranyl acetate and citrate. Observation was performed using a Tecnai G2 Spirit Twin transmission electron microscope (FEI Company, USA) and a JEM ARM 1300S high-voltage electron microscope (JEOL, Japan).

### Intraperitoneal glucose tolerance (IPGTT) and insulin tolerance tests (ITT)

For IPGTT, mice were fasted for 16 h and then 2 g/kg or 1 g/kg glucose was injected into the intraperitoneal cavity of each mouse. Blood glucose levels were measured at 0, 15, 30, 60, and 90 min using a glucometer (Bayer breeze). ITT was performed by measuring blood glucose after 6 h of fasting followed by intraperitoneal insulin injection (0.75 U/kg; Humalog).

### Hyperinsulinemic euglycemic clamping

Hyperinsulinemic euglycemic clamping was performed as previously described [57]. Briefly, after an overnight fast, a 2 h hyperinsulinemic euglycemic clamping was performed in *Crif1^f/+,Fabp4^* and control littermates (*n* = 8). The insulin clamp began with a primed-continuous infusion of insulin (0.3 U/kg bolus followed by 2.5 mU/kg/min). Blood samples (20 µl) were collected at 10 to 20 min intervals for immediate measurement of plasma glucose concentrations, and 20% glucose was infused at variable rates to maintain glucose at basal concentrations (∼120 mg/dL). Basal and insulin-stimulated whole-body glucose uptake was estimated with a continuous infusion of ^3^H glucose (Perkin Elmer Life and Analytical Sciences) for 2 h before clamping (0.05 µCi/min) and throughout the clamping (0.1 µCi/min), respectively. At 75 min after the start of the clamp, 2-deoxy-d-1-^14^C glucose (PerkinElmer Life and Analytical Sciences) was injected with a Hamilton syringe to measure insulin-stimulated glucose transport activity and metabolism in skeletal muscle. Blood samples were taken before, during, and at the end of the clamps for measurement of plasma ^3^H glucose and 2-deoxy-d-1-^14^C glucose concentrations, and/or insulin concentrations. At the end of the clamps, tissue samples (gastrocnemius, eWAT, and liver) were rapidly taken and stored at −70°C prior to biochemical and molecular analysis.

### Flow cytometry

To quantified M1 macrophages, M2 macrophages, and CD4+ and CD8+ T cell populations, the stromal vascular fractions (SVF) was isolated from mouse eWAT. The SVF was prepared by the lysis of eWAT with type 1 collagenase (Gibco BRL) in collagenase buffer at 37°C in a shaking water bath for 40 min, followed by centrifuging at 2000 rpm for 5 min. The suspended solid matter comprised adipocytes and the cell pellet comprised T cells, B cells and macrophages. The cell pellet was then incubated with RBC lysis buffer and the remaining cells were stained with specific antibodies. Anti-CD3 (BD bioscience), anti-CD4 (BD Bioscience) and anti-CD8 (eBioscience) were used to stain the T cell population [58], and F4/80 (eBioscience), CD206 (eBioscience) and CD11c (eBioscience) were used to stain M1/M2 macrophages. The stained SVF cells were analyzed using a FACScan flow cytometer (BD Bioscience) and data analysis was performed using BD FACSDiva software (BD Bioscience).

### Thiobarbituric acid reactive substance (TBAR) assay

The TBAR assay kit (Cayman Chemicals) was used to measure lipid peroxidation in the WAT and plasma of mice. WAT (25 mg) suspended in RIPA buffer was sonicated, centrifuged at 1,600 g for 10 min at 4°C, and the supernatant was collected. The SDS solution was added to the supernatant, which was then mixed with the Color reagent according to the manufacturer's instructions. The sample was boiled for 1 h, centrifuged, and the supernatant was collected. Fluorescence at the excitation wavelength of 530 nm and emission wavelength of 550 nm was measured.

### Macrophage depletion by clodronate

The generation of liposome-encapsulated clodronate was performed as previously described [38]. Cholesterol (10 mg/ml; Sigma-Aldrich) was dissolved in 100% ethanol, and 100 mg/ml phosphatidylcholine in 100% ethanol (Sigma-Aldrich) was made into a phospho-lipid film by drying with a low-vacuum rotary. Clodronate (0.6 M) (Sigma-Aldrich) was dissolved in purified water and incubated with the phospho-lipid film by gentle rotation at room temperature and sonication in a water bath for 3 min at 55 kHz. After removing the non-encapsulated clodronate, liposome-encapsulated clodronate was resuspended in 1X PBS. Two intraperitoneal injections (3 days apart) of clodronate were administered to mice fed a HFD for 8 weeks. IPGTT and ITT were performed 6 days after the first injection.

### Analysis of triglyceride, ceramide, and saturated fatty acids in tissues

Measurement of hepatic triglycerides: Liver triglycerides were extracted with chloroform and methanol, dissolved in 1× PBS, and measured in a Hitachi 7150 chemistry analyzer (Hitachi, Japan).

Measurement of ceramides in WAT, liver and muscle: Prior to extraction of total lipids, C17 ceramide was added as an internal standard. Ceramides were measured as previously described [59]. All liquid chromatography-mass spectrometry (LC-MS/MS) experiments were performed using an Agilent 1200 HPLC system (Agilent Technologies, Santa Clara, CA, USA) coupled to a Thermo LTQ linear ion trap mass spectrometer (Thermo Scientific, San Jose, CA) equipped with an electro spray ionization (ESI) source. Briefly, LC separation was achieved using a LunaC18 RP column (150 mm×2 mm I.D., 5 µm 100 Å particles; Phenomenex, Torrance, CA) with gradient elution. Lipid molecules separated by LC were detected by the mass spectrometer in Positive ESI mode using selected reaction monitoring (SRM). The SRM channels were arranged as follows: 538→264 for C16, 552→264 for C17, 566→264 for C18, 594→264 for C20, 648→264 for C24:1, and 650→264 for C24. The peak area was normalized according to the internal standard and tissue weight. All values are presented as relative differences in the ratio of the extracted lipids to the internal standard.

To measure the level of saturated fatty acids, tissues were homogenized in ice-cold methanol, and 1 µg of pentadecanoic acid (C_15_:0) was added as an internal standard. Samples were incubated at 45°C overnight, then cooled to room temperature. Hexane and 1 mL of H_2_O were added, samples were vortexed and centrifuged, and fatty acid methyl esters were collected from the upper hexane layer. Samples were analyzed by gas chromatography–mass spectrometry (GC-MS) using an Agilent HP6890 GC interfaced with an HP5973N MSD. A DB-5 column was used. The GC oven temperature was initially 150°C and then increased to 280°C for 52 min. Full MS scans over a m/z range of 60 to 800 were obtained and the peaks of the characteristic ion chromatogram for each fatty acid methyl ester were used for quantification. All samples were normalized against the internal standard.

### Serum analysis

Whole cardiac blood from the mice was incubated at room temperature for 2 h. The blood was centrifuged at 2,500 rpm for 5 min, and the supernatant was collected. TNFα and IL-4 were measured using a mouse cytokine/chemokine multiplex panel (Millipore). MCP1 and IP10 were measured using an ELISA kit (R&D Systems). Serum triglycerides and FFAs were measured with a Hitachi 7150 chemistry analyzer (Hitachi, Japan).

### Statistical analyses

Data are presented as means ± or + standard deviation (SD). Statistical significance for comparisons was determined using the Student's two-tailed *T*-test. A p value less than 0.05 was considered statistically significant.

## Supporting Information

Figure S1 Expression of *Crif1* mRNA in the various tissues in control C57BL/6 mouse, *ob/ob* and *db/db* mice. (A) Measurement of *Crif1* mRNA in multiple tissues in 8 weeks-of-aged C57BL/6 male mouse by northern blot analysis. The bars represent the relative density of *Crif1/β-actin* mRNA compared with the value of brain in northern blots. BAT, brown adipose tissue; eWAT, epididymal white adipose tissue; EDL, extensor digitorum longus; GA, gastrocnemius. (B) *Crif1* expression was measured in 7 weeks old *db/db* (left) or *ob/ob* (right) mice and control heterozygous mice by real-time PCR, normalized with 18s ribosomal RNA. (*n* = 6). Values are means + SD, *p<0.05 versus control mice. (C) *Crif1* expression in mice fed a high fat diet (HFD) for 8 weeks starting from 6 weeks of age (*n* = 10). Values are means + SD, *p<0.05 versus control mice. NCD, normal chow diet; HFD, high-fat diet.
(PDF)

Figure S2 Impaired non-shivering thermogenesis in *Crif1^f/f,Fabp4^* mice. All analysis of these mice fed with a NCD was performed at 3 weeks-of-age. (A) Hematoxylin and eosin (H&E) staining of BAT. Scale: 100 µm. (B) Transmission electron microscopy (TEM) of BAT revealed that the mitochondria of *Crif1^f/f,Fabp4^* mice developed severe swollen cristae (red arrows). L, lipid droplet; N, nucleus. Scale: 6,000 nm. (C) Number of mitochondria per area and relative mitochondria size in BAT (n = 20). Values are means + SD, *p<0.05 versus control mice. (D) Body temperature was measured rectally with a digital thermometer at an ambient temperature (23°C) and after emersion in cold water (4°C) for 5 min (*n* = 5). Values are means + SD. *p<0.05 versus control mice. (E) Survival rate of *Crif1^+/+,Fabp4^* and *Crif1^f/f,Fabp4^* mice housed in ambient (23°C) and thermoneutrality (30°C) conditions (*n* = 20).
(PDF)

Figure S3 Levels of mitochondrial OXPHOS complexes in non-adipose tissues of *Crif1^f/+,Fabp4^* mice. (A) Western blotting of Crif1 and the ND1 subunit of OXPHOS complex I in liver and heart. ND1, subunit of OXPHOS complex I. (B) Blue native-PAGE analysis of OXPHOS complexes (I, II, III, IV and V) in mitochondria isolated from liver of *Crif1^+/+,Fabp4^* and *Crif1^f/+,Fabp4^* mice (C) Level of ATP in the heart of *Crif1^+/+,Fabp4^* and *Crif1^f/+,Fabp4^* mice (*n* = 8). Values are means + SD. n.s, not significant.
(PDF)

Figure S4 Adipose development and lipid metabolites in *Crif1^f/+,Fabp4^* mice. *Crif1^+/+,Fabp4^ and Crif1^f/+,Fabp4^* mice were generated from floxed *Crif1* mice with Fabp4-*Cre* recombinase mice. NCD or a HFD were begun at 6 weeks-of-age. (A) Body weight changes in mice fed a NCD or 60% HFD for 8 weeks or 14 weeks, starting at 6 weeks-of-age (*n* = 8). Values are means + SD. wks, weeks; n.s, not significant. (B) Daily food intake of *Crif1^+/+,Fabp4^* and *Crif1^f/+,Fabp4^* mice (*n* = 8). (*Crif1^+/+,Fabp4^*, 3.24+0.42 g/day *vs Crif1^f/+,Fabp4^*, 2.94+0.14 g/day) Values are means + SD. n.s, not significant. (C) MR images in *Crif1^+/+,Fabp4^* and *Crif1^f/+,Fabp4^* mice fed with NCD and HFD for 14 weeks. (D and E) Serum TG and FFA levels in control (*Crif1^+/+,Fabp4^*) and adipose tissue-specific *Crif1* heterozygous mice (*Crif1^f/+,Fabp4^*) (*n* = 8). Values are means + SD, *p<0.05, n.s, not significant. (F) TG levels in the liver. The peak area was normalized according to a liver weight (*n* = 8). Values are means + SD, *p<0.05 versus control mice. (G) Combined level of saturated fatty acids such as C16, C18, C18.1, C18.3, and C20.4 in WAT, gastrocnemius muscle and liver quantified by gas chromatography–mass spectrometry (GC-MS). The data was normalized according to an internal standard and tissue wet weight (*n* = 4). Values are means + SD. n.s, not significant. (H) Combined level of ceramides, such as C16, C18, C20, C22, C24 and C24.1 in the WAT, gastrocnemius muscle, and liver of mice fed a HFD for 14 weeks quantified by liquid chromatography-mass spectrometry (LC-MS/MS). The peak area was normalized according to an internal standard (C17 ceramide) and tissue weight (*n* = 4). Values are means + SD. n.s, not significant.
(PDF)

Figure S5 Gene expression profiles in 3T3-L1 adipocytes following silencing of *Crif1* determined by a complementary DNA microarray. A microarray was performed using 3T3-L1 adipocytes treated with control or *Crif1* siRNA with Agilent's DNA microarray Chip. Data were analyzed using the Feature Extraction and GeneSpring Software (Agilent Technologies).
(PDF)

Figure S6 Activation of p38 MAPK and expression of chemokines in MEFs. (A) p-p38 MAPK and t-p38 MAPK levels in control (+/Δ) and *Crif1*-null (−/Δ) MEFs. p38 MAPK, p38 mitogen-activated protein kinases. (B) Real-time PCR with *Mcp1* and *Ip10* primers in MEF null cells (−/Δ) (*n* = 6). Values are means + SD. n.s, not significant.
(PDF)

Figure S7 ROS and chemokine secretion in mice. (A) Measurement of lipid peroxidation with the TBAR assay in the WAT and plasma of *Crif1^+/+,Fabp4^* and *Crif1^f/+,Fabp4^* mice fed a NCD or HFD for 8 weeks (*n* = 8). Values are means + SD. *p<0.05, n.s, not significant. (B) Secreted MCP1 and IP10 levels in the serum of *Crif1^+/+,Fabp4^* and *Crif1^f/+,Fabp4^* mice fed a NCD or HFD for 8 weeks (*n* = 8). Values are means + SD. *p<0.05, n.s, not significant.
(PDF)

Figure S8 Phenotypic analysis and detection of *Cre*-recombinase activity in peritoneal macrophages from *Crif1^+/+,Fabp4^* and *Crif1^f/+,Fabp4^* mice. (A) Peritoneal macrophages were collected following intraperitoneal injection of thioglycollate into 6-week-old *Crif1^+/+,Fabp4^* and *Crif1^f/+,Fabp4^* mice. Expression of *Crif1* and phenotypic markers for M1 and M2 macrophages were measured by real-time PCR using specific primers (*n* = 10). Values are means + SD. *p<0.05, n.s, not significant. (B) Schematic showing the loxp site in the *Crif1* gene and the location of primers 1, 2, and 3. (C) PCR analysis of homologous recombination in genomic DNA extracted from WAT, BAT, and peritoneal macrophages isolated from 20-week-old control and *Crif1^f/+,Fabp4^* mice. Genomic DNA for *Cre* recombinase was detected in WAT, BAT, and peritoneal macrophages (upper panel), but the recombination product was only present in WAT and BAT (lower panel).
(PDF)

Figure S9 Generation of adipocyte specific *Crif1* knockout mouse with Adipoq-*Cre* mice. (A) All analysis of these mice fed with a NCD was performed at 8 weeks-of-age. *Crif1* mRNA levels in eWAT and BAT of control (*Crif1^+/+,Adipoq^*), adipose-specific *Crif1* heterozygous (*Crif1^f/+,Adipoq^*), and homozygous (*Crif1^f/f,Adipoq^*) knockout mice (*n* = 6). Values are means + SD. *p<0.05 versus the control mice. (B) Western blot analysis of Crif1, subunit of OXPHOX complex I (ND1 and NDUFA9), OXPHOX complex III (UQCRC2), OXPHOX complex IV (COX4) in eWAT, BAT, heart and liver from the three strains of mice. (C) The body temperature of 3-week-old mice was measured when exposed to an ambient temperature (23°C) and after emersion in cold water (4°C) for 5 min (n = 5). Values are means + SD. *p<0.05 versus control mice, n.s, not significant.
(PDF)

Figure S10 Metabolic phenotypes of liver specific- or skeletal muscle specific-*Crif1* KO mice. (A and B) Body weight and daily food intake of control (*Crif1^+/+,Alb^*) and liver specific-*Crif1* homozygous KO mice (*Crif1^f/f,Alb^*) fed a HFD diet for 8 weeks (*n* = 8). Values are means + SD. n.s, not significant. (C) IPGTT experiment with *Crif1^+/+,Alb^* and *Crif1^f/f,Alb^* mice injected with 1 g/kg glucose after 16 h of fasting (*n* = 8). Values are means ± SD. (D) Real time PCR with specific primers to measure gluconeogenic gene expression in the livers of fasted mice. (E) Body weight of control (*Crif1^+/+,MLC^*) and skeletal muscle-specific *Crif1* haploinsufficientt mice (*Crif1^f/+,MLC^*) fed a NCD diet. Values are means + SD. n.s, not significant. (F) IPGTT experiment with *Crif1^f/+,MLC^* mice injected with 2 g/kg glucose after 16 h of fasting (*n* = 8). Values are means ± SD.
(PDF)

Table S1 Summary of the phenotypes of adipocyte-specific *Crif1* mutant mice under the control of the Fabp4-*Cre* or Adipoq-*Cre* promoters. The percentages represent residual levels in comparison to control mice.* The band intensities of Crif1 protein were determined by Western blot analysis using 6 male mice per group. ** Adipose mass was evaluated by totaling the weights of eWAT of 6 male mice per group. n.d; not determined.
(DOCX)

Table S2 Sequence of primers used in real-time PCR.
(DOCX)

## References

[1] Rosen ED, Spiegelman BM (2006) *Adipocytes as regulators of energy balance and glucose homeostasis* . Nature 444 (7121) 847–53. 17167472 10.1038/nature05483 PMC3212857

[2] Flier JS (2004) Obesity wars: molecular progress confronts an expanding epidemic. Cell 116 (2) 337–50. 14744442 10.1016/s0092-8674(03)01081-x

[3] Kahn BB, Flier JS (2000) *Obesity and insulin resistance* . J Clin Invest 106 (4) 473–81. 10953022 10.1172/JCI10842 PMC380258

[4] Hotamisligil GS (2010) Endoplasmic reticulum stress and the inflammatory basis of metabolic disease. Cell 140 (6) 900–17. 20303879 10.1016/j.cell.2010.02.034 PMC2887297

[5] Kusminski CM, Scherer PE (2012) *Mitochondrial dysfunction in white adipose tissue* . Trends Endocrinol Metab 23 (9) 435–43. 22784416 10.1016/j.tem.2012.06.004 PMC3430798

[6] Patti ME, Corvera S (2010) *The role of mitochondria in the pathogenesis of type 2 diabetes* . Endocr Rev 31 (3) 364–95. 20156986 10.1210/er.2009-0027 PMC3365846

[7] Tormos KV, et al (2011) Mitochondrial complex III ROS regulate adipocyte differentiation. Cell Metab 14 (4) 537–44. 21982713 10.1016/j.cmet.2011.08.007 PMC3190168

[8] Shi X, et al (2008) Paradoxical effect of mitochondrial respiratory chain impairment on insulin signaling and glucose transport in adipose cells. J Biol Chem 283 (45) 30658–67. 18779333 10.1074/jbc.M800510200 PMC2576555

[9] Koh EH, et al (2007) Essential role of mitochondrial function in adiponectin synthesis in adipocytes. Diabetes 56 (12) 2973–81. 17827403 10.2337/db07-0510

[10] Wilson-Fritch L, et al (2004) Mitochondrial remodeling in adipose tissue associated with obesity and treatment with rosiglitazone. J Clin Invest 114 (9) 1281–9. 15520860 10.1172/JCI21752 PMC524228

[11] Rong JX, et al (2007) Adipose mitochondrial biogenesis is suppressed in db/db and high-fat diet-fed mice and improved by rosiglitazone. Diabetes 56 (7) 1751–60. 17456854 10.2337/db06-1135

[12] Kaaman M, et al (2007) Strong association between mitochondrial DNA copy number and lipogenesis in human white adipose tissue. Diabetologia 50 (12) 2526–33. 17879081 10.1007/s00125-007-0818-6

[13] Dahlman I, et al (2006) Downregulation of electron transport chain genes in visceral adipose tissue in type 2 diabetes independent of obesity and possibly involving tumor necrosis factor-alpha. Diabetes 55 (6) 1792–9. 16731844 10.2337/db05-1421

[14] Wilson-Fritch L, et al (2003) Mitochondrial biogenesis and remodeling during adipogenesis and in response to the insulin sensitizer rosiglitazone. Mol Cell Biol 23 (3) 1085–94. 12529412 10.1128/MCB.23.3.1085-1094.2003 PMC140688

[15] Bogacka I, et al (2005) Pioglitazone induces mitochondrial biogenesis in human subcutaneous adipose tissue in vivo. Diabetes 54 (5) 1392–9. 15855325 10.2337/diabetes.54.5.1392

[16] Tedesco L, et al (2008) Cannabinoid type 1 receptor blockade promotes mitochondrial biogenesis through endothelial nitric oxide synthase expression in white adipocytes. Diabetes 57 (8) 2028–36. 18477809 10.2337/db07-1623 PMC2494670

[17] Kim SJ, et al (2012) CRIF1 is essential for the synthesis and insertion of oxidative phosphorylation polypeptides in the mammalian mitochondrial membrane. Cell Metab 16 (2) 274–83. 22819524 10.1016/j.cmet.2012.06.012

[18] Kwon MC, et al (2008) *Crif1 is a novel transcriptional coactivator of STAT3* . EMBO J 27 (4) 642–53. 18200042 10.1038/sj.emboj.7601986 PMC2262042

[19] Urs S, et al (2006) Selective expression of an aP2/Fatty Acid Binding Protein 4-Cre transgene in non-adipogenic tissues during embryonic development. Transgenic Res 15 (5) 647–53. 16952017 10.1007/s11248-006-9000-z

[20] Xue B, et al (2007) Genetic variability affects the development of brown adipocytes in white fat but not in interscapular brown fat. J Lipid Res 48 (1) 41–51. 17041251 10.1194/jlr.M600287-JLR200

[21] Pospisilik JA, et al (2007) Targeted deletion of AIF decreases mitochondrial oxidative phosphorylation and protects from obesity and diabetes. Cell 131 (3) 476–91. 17981116 10.1016/j.cell.2007.08.047

[22] Rocha VZ, Libby (2009) *Obesity, inflammation, and atherosclerosis* . Nat Rev Cardiol 6 (6) 399–409. 19399028 10.1038/nrcardio.2009.55

[23] Sun K, Kusminski CM, Scherer PE (2011) *Adipose tissue remodeling and obesity* . J Clin Invest 121 (6) 2094–101. 21633177 10.1172/JCI45887 PMC3104761

[24] Gao YJ, et al (2009) JNK-induced MCP-1 production in spinal cord astrocytes contributes to central sensitization and neuropathic pain. J Neurosci 29 (13) 4096–108. 19339605 10.1523/JNEUROSCI.3623-08.2009 PMC2682921

[25] Shen Q, Zhang R, Bhat NR (2006) MAP kinase regulation of IP10/CXCL10 chemokine gene expression in microglial cells. Brain Research 1086: 9–16. 16635481 10.1016/j.brainres.2006.02.116

[26] Shoelson SE, Lee J, Goldfine AB (2006) *Inflammation and insulin resistance* . J Clin Invest 116 (7) 1793–801. 16823477 10.1172/JCI29069 PMC1483173

[27] Chen CL, et al (2008) Ceramide induces p38 MAPK and JNK activation through a mechanism involving a thioredoxin-interacting protein-mediated pathway. Blood 111 (8) 4365–74. 18270325 10.1182/blood-2007-08-106336

[28] Olefsky JM, Glass CK (2010) *Macrophages, inflammation, and insulin resistance* . Annu Rev Physiol 72: 219–46. 20148674 10.1146/annurev-physiol-021909-135846

[29] Samuel VT, Shulman GI (2012) Mechanisms for insulin resistance: common threads and missing links. Cell 148 (5) 852–71. 22385956 10.1016/j.cell.2012.02.017 PMC3294420

[30] Kanda H, et al (2006) MCP-1 contributes to macrophage infiltration into adipose tissue, insulin resistance, and hepatic steatosis in obesity. J Clin Invest 116 (6) 1494–505. 16691291 10.1172/JCI26498 PMC1459069

[31] Lumeng CN, Maillard I, Saltiel AR (2009) *T-ing up inflammation in fat* . Nat Med 15 (8) 846–7. 19661987 10.1038/nm0809-846

[32] Nishimura S, et al (2009) CD8+ effector T cells contribute to macrophage recruitment and adipose tissue inflammation in obesity. Nat Med 15 (8) 914–20. 19633658 10.1038/nm.1964

[33] Hotamisligil GS, et al (1995) Increased adipose tissue expression of tumor necrosis factor-alpha in human obesity and insulin resistance. J Clin Invest 95 (5) 2409–15. 7738205 10.1172/JCI117936 PMC295872

[34] Schenk S, Saberi M, Olefsky JM (2008) *Insulin sensitivity: modulation by nutrients and inflammation* . J Clin Invest 118 (9) 2992–3002. 18769626 10.1172/JCI34260 PMC2522344

[35] Makowski L, et al (2001) Lack of macrophage fatty-acid-binding protein aP2 protects mice deficient in apolipoprotein E against atherosclerosis. Nat Med 7 (6) 699–705. 11385507 10.1038/89076 PMC4027052

[36] Gareus R, et al (2008) Endothelial cell-specific NF-kappaB inhibition protects mice from atherosclerosis. Cell Metab 8 (5) 372–83. 19046569 10.1016/j.cmet.2008.08.016

[37] Eguchi J, et al (2011) *Transcriptional control of adipose lipid handling by IRF4* . Cell Metab 13 (3) 249–59. 21356515 10.1016/j.cmet.2011.02.005 PMC3063358

[38] van Rooijen N, Hendrikx E (2010) *Liposomes for specific depletion of macrophages from organs and tissues* . Methods Mol Biol 605: 189–203. 20072882 10.1007/978-1-60327-360-2_13

[39] Lowell BB, Shulman GI (2005) *Mitochondrial dysfunction and type 2 diabetes* . Science 307 (5708) 384–7. 15662004 10.1126/science.1104343

[40] Morino K, Petersen KF, Shulman GI (2006) Molecular mechanisms of insulin resistance in humans and their potential links with mitochondrial dysfunction. Diabetes 55 Suppl 2: S9–S15. 17130651 10.2337/db06-S002 PMC2995546

[41] Schmid AI, et al (2011) Liver ATP synthesis is lower and relates to insulin sensitivity in patients with type 2 diabetes. Diabetes Care 34 (2) 448–53. 21216854 10.2337/dc10-1076 PMC3024365

[42] Freyer C, Larsson NG (2007) *Is energy deficiency good in moderation?* . Cell 131 (3) 448–50. 17981112 10.1016/j.cell.2007.10.027

[43] Wredenberg A, et al (2006) Respiratory chain dysfunction in skeletal muscle does not cause insulin resistance. Biochem Biophys Res Commun 350 (1) 202–7. 16996481 10.1016/j.bbrc.2006.09.029

[44] Crunkhorn S, et al (2007) Peroxisome proliferator activator receptor gamma coactivator-1 expression is reduced in obesity: potential pathogenic role of saturated fatty acids and p38 mitogen-activated protein kinase activation. J Biol Chem 282 (21) 15439–50. 17416903 10.1074/jbc.M611214200

[45] Sutherland LN, et al (2008) Time course of high-fat diet-induced reductions in adipose tissue mitochondrial proteins: potential mechanisms and the relationship to glucose intolerance. Am J Physiol Endocrinol Metab 295 (5) E1076–83. 18780775 10.1152/ajpendo.90408.2008

[46] Newton BW, et al (2011) Proteomic analysis of 3T3-L1 adipocyte mitochondria during differentiation and enlargement. J Proteome Res 10 (10) 4692–702. 21815628 10.1021/pr200491h

[47] Lu RH, et al (2010) Mitochondrial development and the influence of its dysfunction during rat adipocyte differentiation. Mol Biol Rep 37 (5) 2173–82. 19693701 10.1007/s11033-009-9695-z

[48] Herrero L, et al (2010) *Inflammation and adipose tissue macrophages in lipodystrophic mice* . Proc Natl Acad Sci U S A 107 (1) 240–5. 20007767 10.1073/pnas.0905310107 PMC2806777

[49] Petersen KF, et al (2004) Impaired mitochondrial activity in the insulin-resistant offspring of patients with type 2 diabetes. N Engl J Med 350 (7) 664–71. 14960743 10.1056/NEJMoa031314 PMC2995502

[50] Osborn O, Olefsky JM (2012) The cellular and signaling networks linking the immune system and metabolism in disease. Nat Med 18 (3) 363–74. 22395709 10.1038/nm.2627

[51] Xu H, et al (2003) Chronic inflammation in fat plays a crucial role in the development of obesity-related insulin resistance. J Clin Invest 112 (12) 1821–30. 14679177 10.1172/JCI19451 PMC296998

[52] Weisberg SP, et al (2003) *Obesity is associated with macrophage accumulation in adipose tissue* . J Clin Invest 112 (12) 1796–808. 14679176 10.1172/JCI19246 PMC296995

[53] Lumeng CN, Bodzin JL, Saltiel AR (2007) *Obesity induces a phenotypic switch in adipose tissue macrophage polarization* . J Clin Invest 117 (1) 175–84. 17200717 10.1172/JCI29881 PMC1716210

[54] Lee KW, et al (2011) Effects of Sulfonylureas on Peroxisome Proliferator-Activated Receptor gamma Activity and on Glucose Uptake by Thiazolidinediones. Diabetes Metab J 35 (4) 340–7. 21977453 10.4093/dmj.2011.35.4.340 PMC3178694

[55] Estes BT, et al (2010) Isolation of adipose-derived stem cells and their induction to a chondrogenic phenotype. Nat Protoc 5 (7) 1294–311. 20595958 10.1038/nprot.2010.81 PMC3219531

[56] Boldogh IR, Pon LA (2007) Purification and subfractionation of mitochondria from the yeast Saccharomyces cerevisiae. Methods Cell Biol 80: 45–64. 17445688 10.1016/S0091-679X(06)80002-6

[57] Park SY, et al (2005) Cardiac-specific overexpression of peroxisome proliferator-activated receptor-alpha causes insulin resistance in heart and liver. Diabetes 54 (9) 2514–24. 16123338 10.2337/diabetes.54.9.2514

[58] Kang SM, et al (2008) CD4+CD25+ regulatory T cells selectively diminish systemic autoreactivity in arthritic K/BxN mice. Mol Cells 25 (1) 64–9. 18319615

[59] Kasumov T, et al (2010) Quantification of ceramide species in biological samples by liquid chromatography electrospray ionization tandem mass spectrometry. Anal Biochem 401 (1) 154–61. 20178771 10.1016/j.ab.2010.02.023 PMC2872137

