# Supplementary figures and images for: Crif1 Deficiency Reduces Adipose OXPHOS Capacity and Triggers Inflammation and Insulin Resistance in Mice
Source: PLoS Genet. 2013 Mar 14;9(3):e1003356. doi: 10.1371/journal.pgen.1003356 (PMC3597503; doi:10.1371/journal.pgen.1003356)

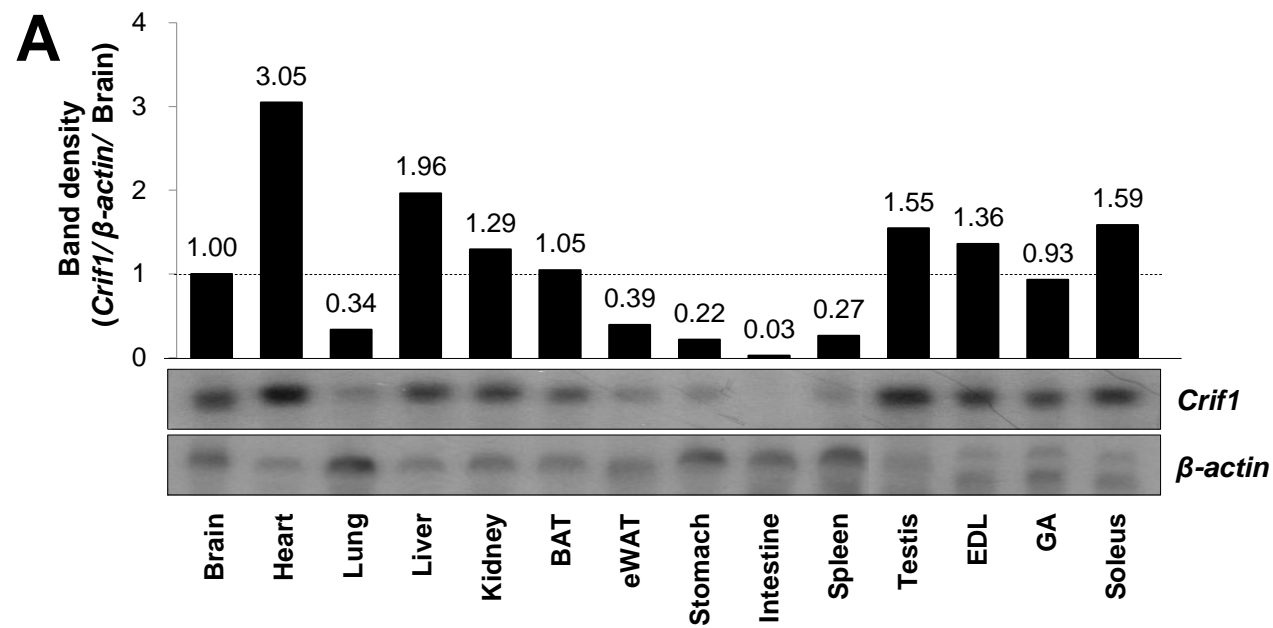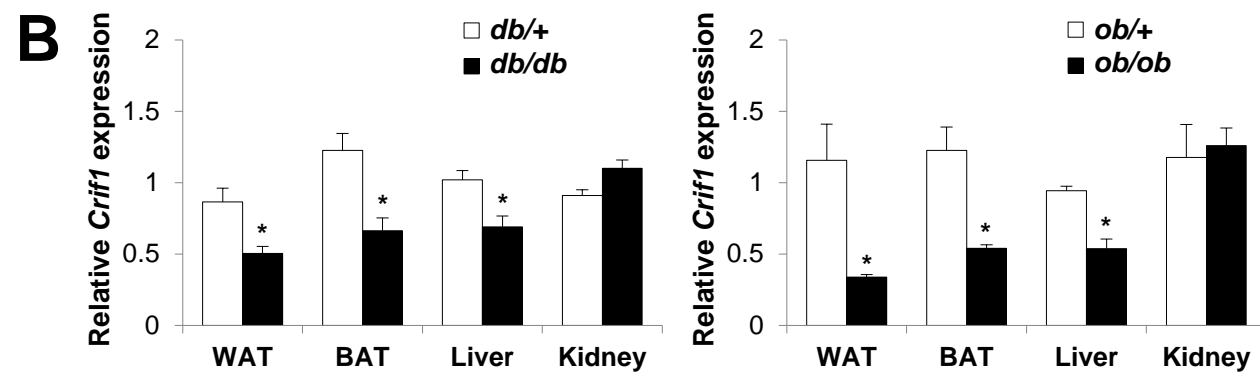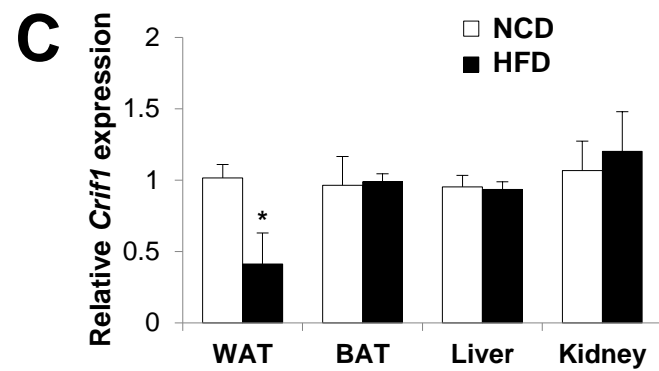

Supplement: Figure S1 — Expression of Crif1 mRNA in the various tissues in control C57BL/6 mouse, ob/ob and db/db mice. (A) Measurement of Crif1 mRNA in multiple tissues in 8 weeks-of-aged C57BL/6 male mouse by northern blot analysis. The bars represent the relative density of Crif1/β-actin mRNA compared with the value of brain in northern blots. BAT, brown adipose tissue; eWAT, epididymal white adipose tissue; EDL, extensor digitorum longus; GA, gastrocnemius. (B) Crif1 expression was measured in 7 weeks old db/db (left) or ob/ob (right) mice and control heterozygous mice by real-time PCR, normalized with 18s ribosomal RNA. (n = 6). Values are means + SD, *p<0.05 versus control mice. (C) Crif1 expression in mice fed a high fat diet (HFD) for 8 weeks starting from 6 weeks of age (n = 10). Values are means + SD, *p<0.05 versus control mice. NCD, normal chow diet; HFD, high-fat diet. (PDF) [file pgen.1003356.s001.pdf]

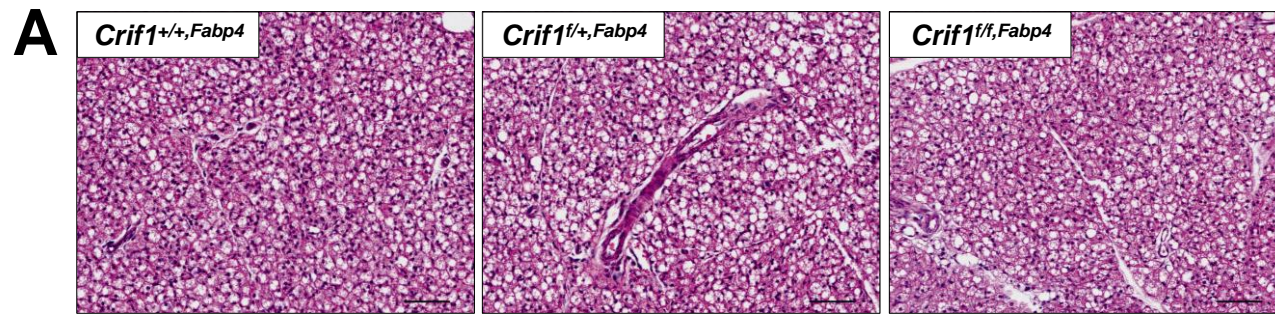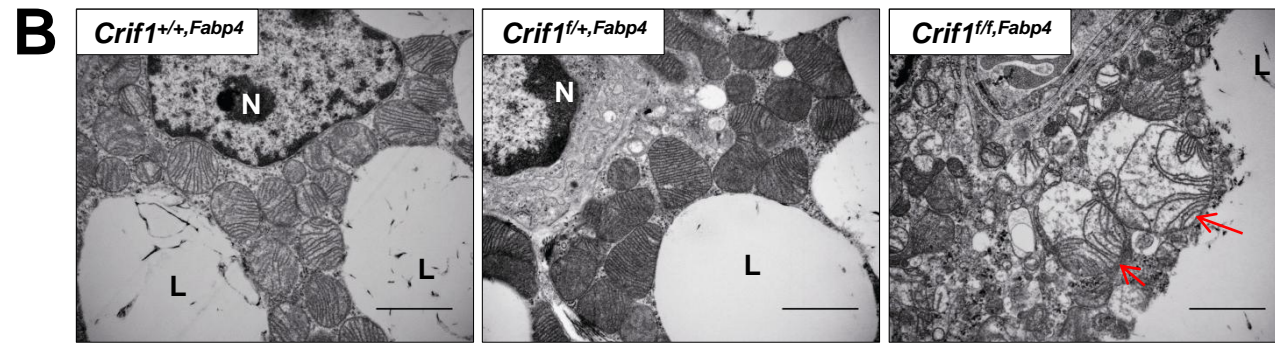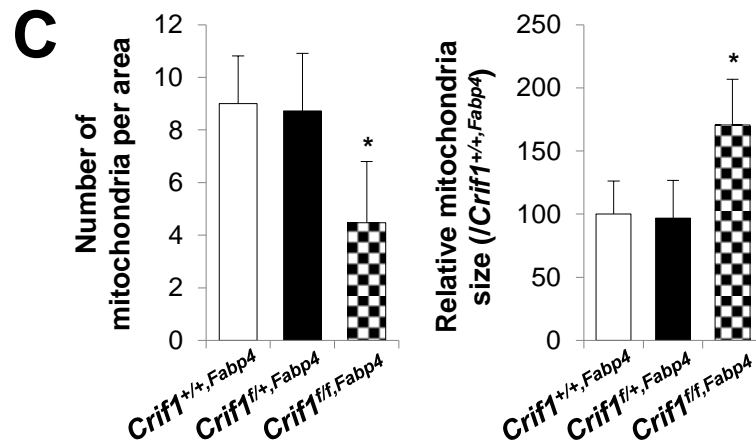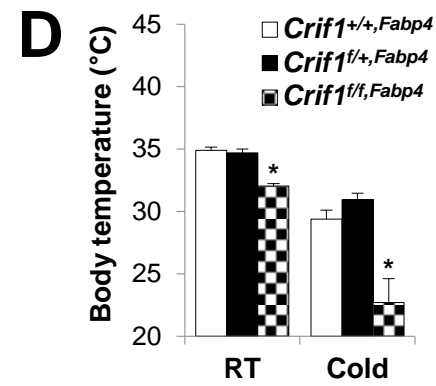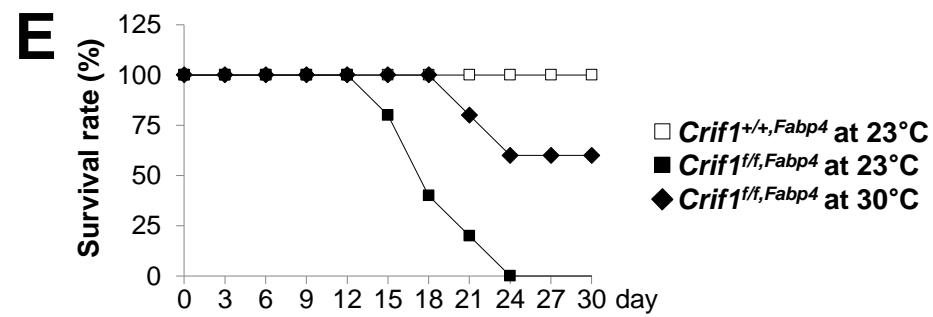

Supplement: Figure S2 — Impaired non-shivering thermogenesis in Crif1f/f,Fabp4 mice. All analysis of these mice fed with a NCD was performed at 3 weeks-of-age. (A) Hematoxylin and eosin (H&E) staining of BAT. Scale: 100 µm. (B) Transmission electron microscopy (TEM) of BAT revealed that the mitochondria of Crif1f/f,Fabp4 mice developed severe swollen cristae (red arrows). L, lipid droplet; N, nucleus. Scale: 6,000 nm. (C) Number of mitochondria per area and relative mitochondria size in BAT (n = 20). Values are means + SD, *p<0.05 versus control mice. (D) Body temperature was measured rectally with a digital thermometer at an ambient temperature (23°C) and after emersion in cold water (4°C) for 5 min (n = 5). Values are means + SD. *p<0.05 versus control mice. (E) Survival rate of Crif1+/+,Fabp4 and Crif1f/f,Fabp4 mice housed in ambient (23°C) and thermoneutrality (30°C) conditions (n = 20). (PDF) [file pgen.1003356.s002.pdf]

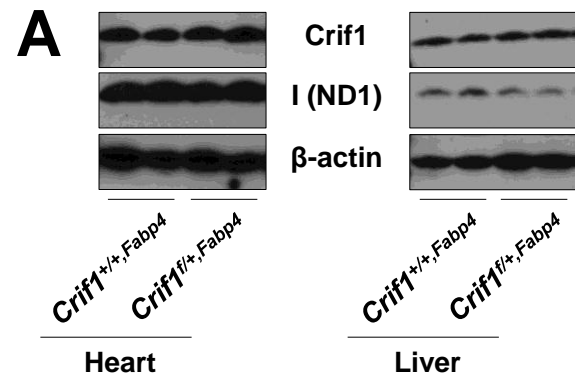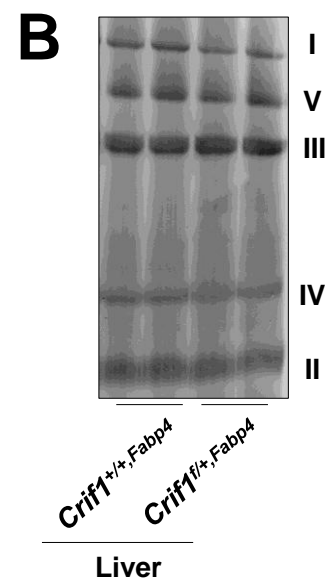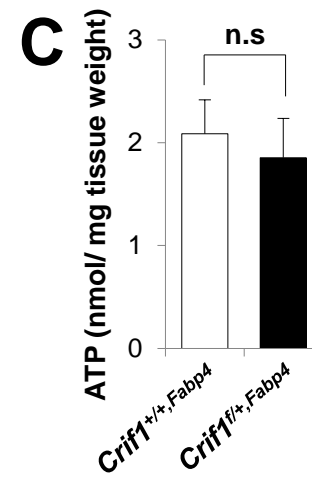

Supplement: Figure S3 — Levels of mitochondrial OXPHOS complexes in non-adipose tissues of Crif1f/+,Fabp4 mice. (A) Western blotting of Crif1 and the ND1 subunit of OXPHOS complex I in liver and heart. ND1, subunit of OXPHOS complex I. (B) Blue native-PAGE analysis of OXPHOS complexes (I, II, III, IV and V) in mitochondria isolated from liver of Crif1+/+,Fabp4 and Crif1f/+,Fabp4 mice (C) Level of ATP in the heart of Crif1+/+,Fabp4 and Crif1f/+,Fabp4 mice (n = 8). Values are means + SD. n.s, not significant. (PDF) [file pgen.1003356.s003.pdf]

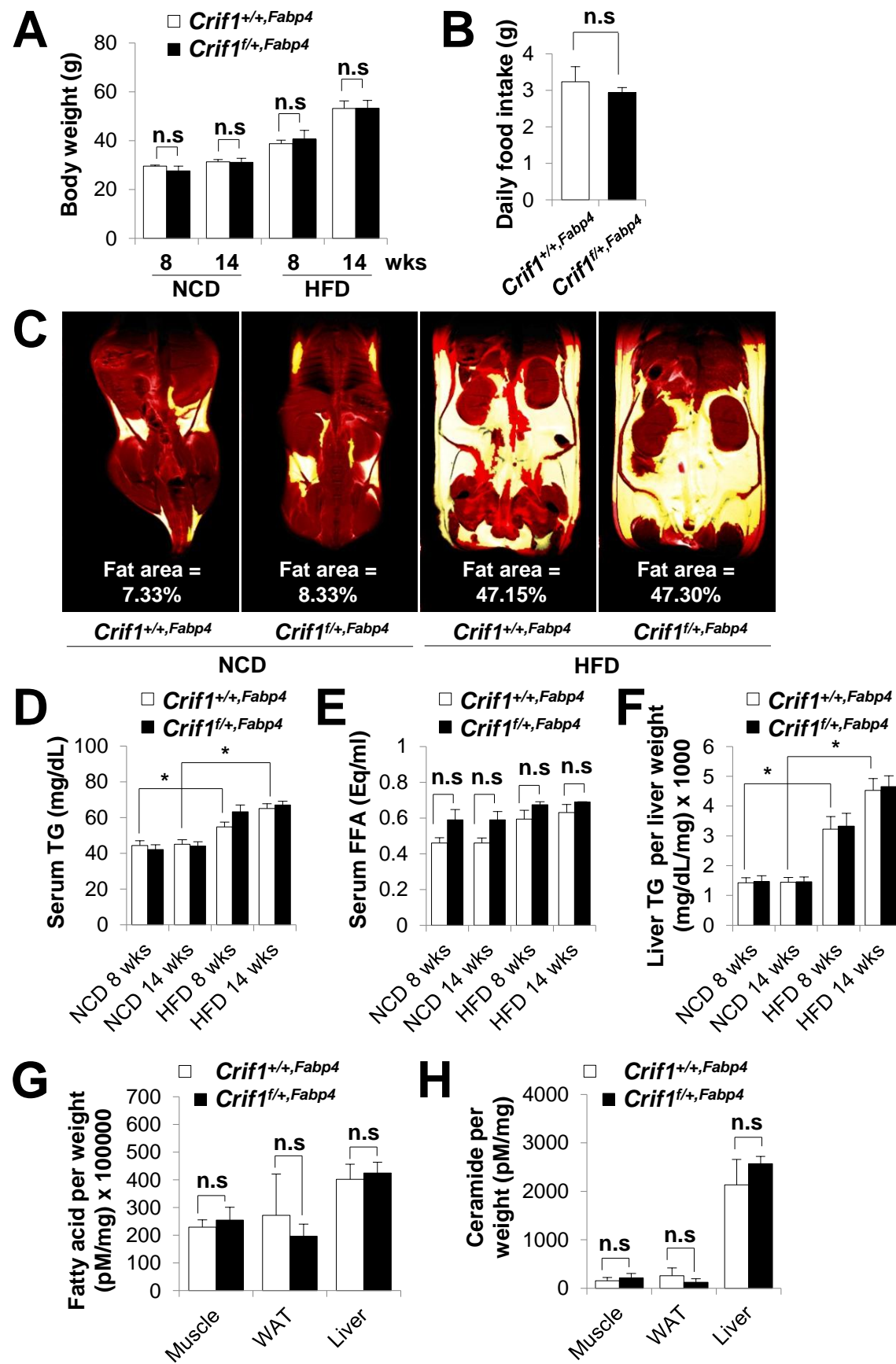

Supplement: Figure S4 — Adipose development and lipid metabolites in Crif1f/+,Fabp4 mice. Crif1+/+,Fabp4 and Crif1f/+,Fabp4 mice were generated from floxed Crif1 mice with Fabp4-Cre recombinase mice. NCD or a HFD were begun at 6 weeks-of-age. (A) Body weight changes in mice fed a NCD or 60% HFD for 8 weeks or 14 weeks, starting at 6 weeks-of-age (n = 8). Values are means + SD. wks, weeks; n.s, not significant. (B) Daily food intake of Crif1+/+,Fabp4 and Crif1f/+,Fabp4 mice (n = 8). (Crif1+/+,Fabp4, 3.24+0.42 g/day vs Crif1f/+,Fabp4, 2.94+0.14 g/day) Values are means + SD. n.s, not significant. (C) MR images in Crif1+/+,Fabp4 and Crif1f/+,Fabp4 mice fed with NCD and HFD for 14 weeks. (D and E) Serum TG and FFA levels in control (Crif1+/+,Fabp4) and adipose tissue-specific Crif1 heterozygous mice (Crif1f/+,Fabp4) (n = 8). Values are means + SD, *p<0.05, n.s, not significant. (F) TG levels in the liver. The peak area was normalized according to a liver weight (n = 8). Values are means + SD, *p<0.05 versus control mice. (G) Combined level of saturated fatty acids such as C16, C18, C18.1, C18.3, and C20.4 in WAT, gastrocnemius muscle and liver quantified by gas chromatography–mass spectrometry (GC-MS). The data was normalized according to an internal standard and tissue wet weight (n = 4). Values are means + SD. n.s, not significant. (H) Combined level of ceramides, such as C16, C18, C20, C22, C24 and C24.1 in the WAT, gastrocnemius muscle, and liver of mice fed a HFD for 14 weeks quantified by liquid chromatography-mass spectrometry (LC-MS/MS). The peak area was normalized according to an internal standard (C17 ceramide) and tissue weight (n = 4). Values are means + SD. n.s, not significant. (PDF) [file pgen.1003356.s004.pdf]

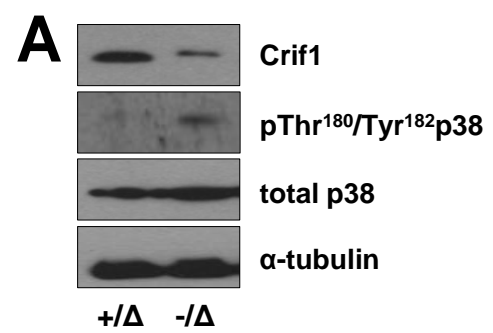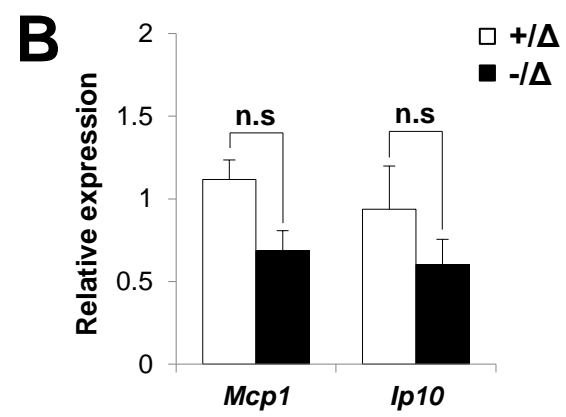

Supplement: Figure S6 — Activation of p38 MAPK and expression of chemokines in MEFs. (A) p-p38 MAPK and t-p38 MAPK levels in control (+/Δ) and Crif1-null (−/Δ) MEFs. p38 MAPK, p38 mitogen-activated protein kinases. (B) Real-time PCR with Mcp1 and Ip10 primers in MEF null cells (−/Δ) (n = 6). Values are means + SD. n.s, not significant. (PDF) [file pgen.1003356.s006.pdf]

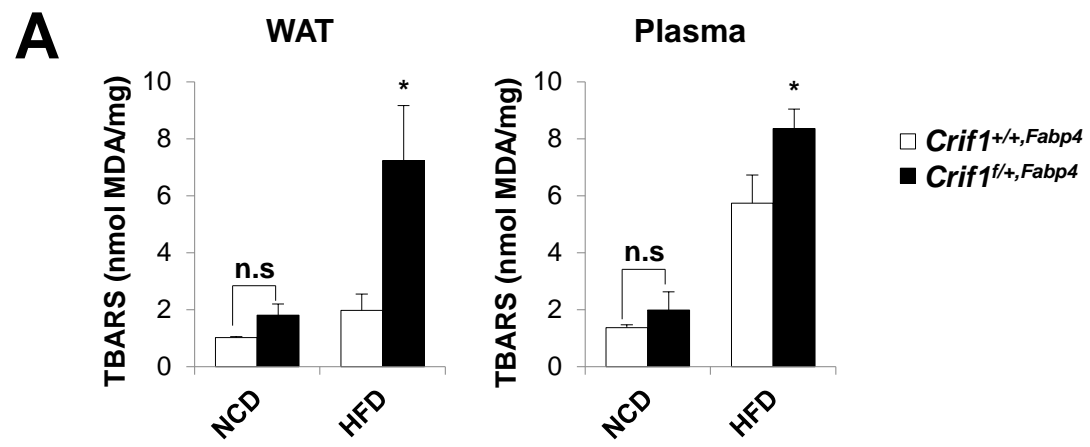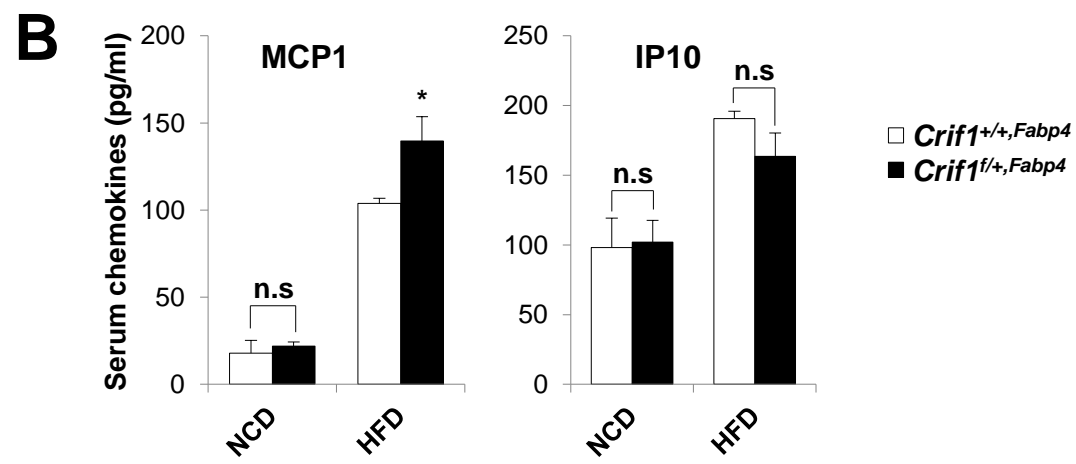

Supplement: Figure S7 — ROS and chemokine secretion in mice. (A) Measurement of lipid peroxidation with the TBAR assay in the WAT and plasma of Crif1+/+,Fabp4 and Crif1f/+,Fabp4 mice fed a NCD or HFD for 8 weeks (n = 8). Values are means + SD. *p<0.05, n.s, not significant. (B) Secreted MCP1 and IP10 levels in the serum of Crif1+/+,Fabp4 and Crif1f/+,Fabp4 mice fed a NCD or HFD for 8 weeks (n = 8). Values are means + SD. *p<0.05, n.s, not significant. (PDF) [file pgen.1003356.s007.pdf]

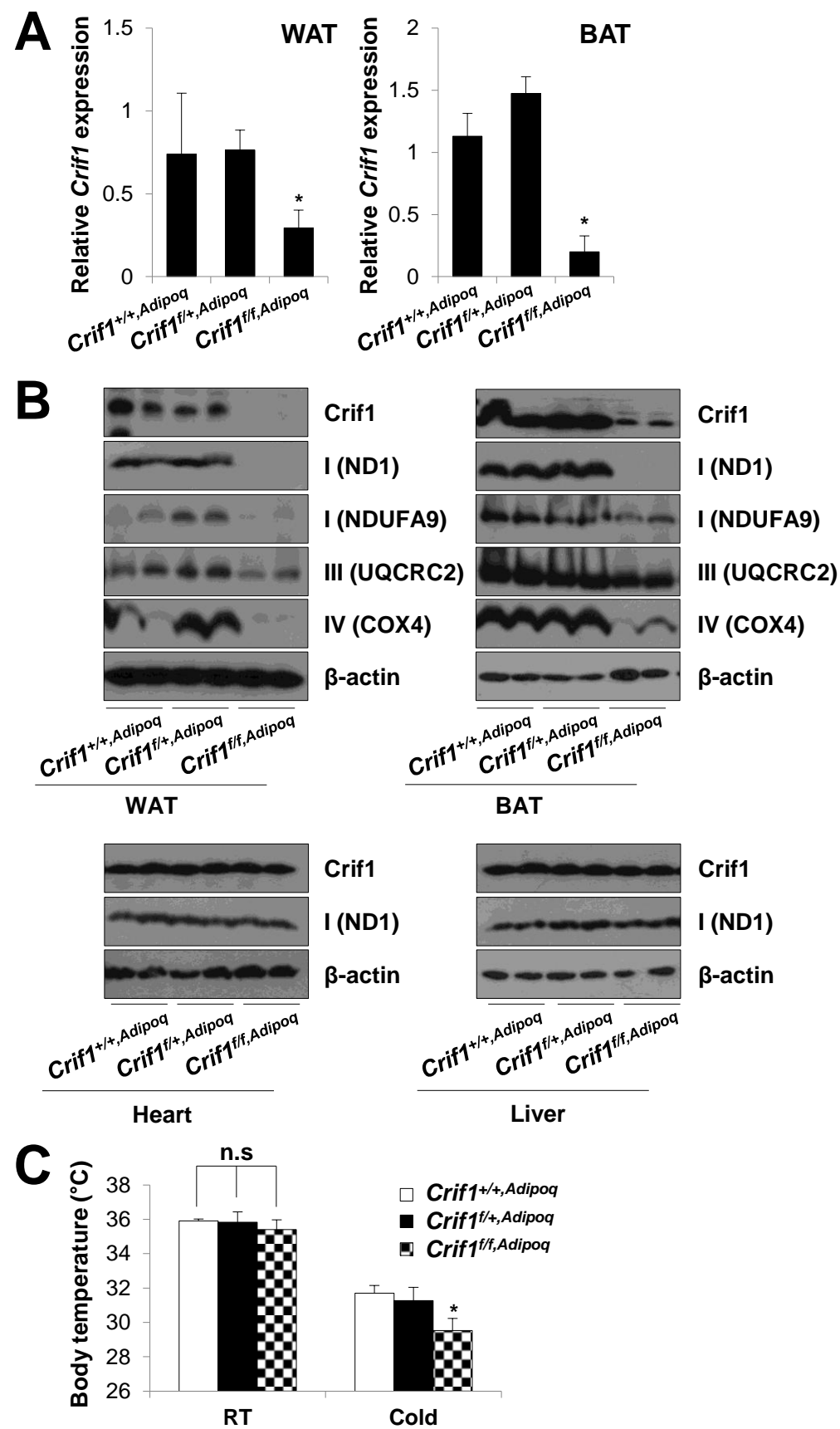

Supplement: Figure S9 — Generation of adipocyte specific Crif1 knockout mouse with Adipoq-Cre mice. (A) All analysis of these mice fed with a NCD was performed at 8 weeks-of-age. Crif1 mRNA levels in eWAT and BAT of control (Crif1+/+,Adipoq), adipose-specific Crif1 heterozygous (Crif1f/+,Adipoq), and homozygous (Crif1f/f,Adipoq) knockout mice (n = 6). Values are means + SD. *p<0.05 versus the control mice. (B) Western blot analysis of Crif1, subunit of OXPHOX complex I (ND1 and NDUFA9), OXPHOX complex III (UQCRC2), OXPHOX complex IV (COX4) in eWAT, BAT, heart and liver from the three strains of mice. (C) The body temperature of 3-week-old mice was measured when exposed to an ambient temperature (23°C) and after emersion in cold water (4°C) for 5 min (n = 5). Values are means + SD. *p<0.05 versus control mice, n.s, not significant. (PDF) [file pgen.1003356.s009.pdf]

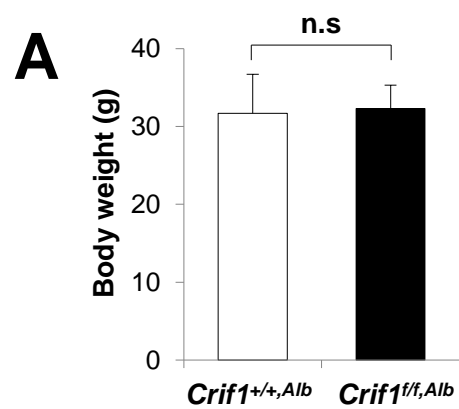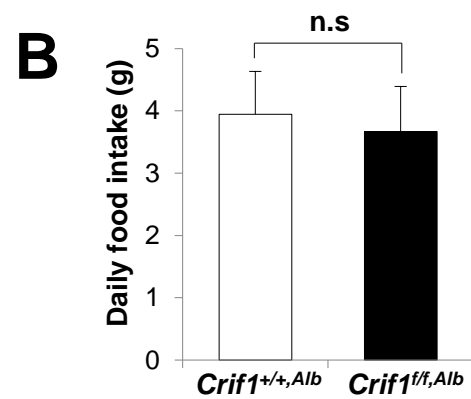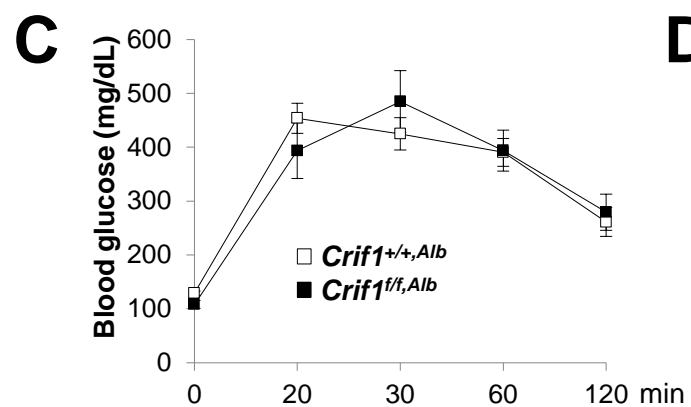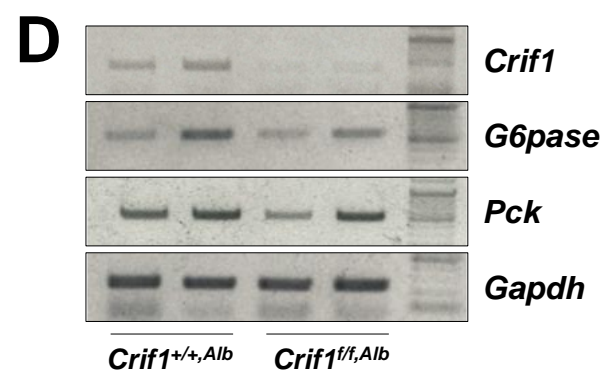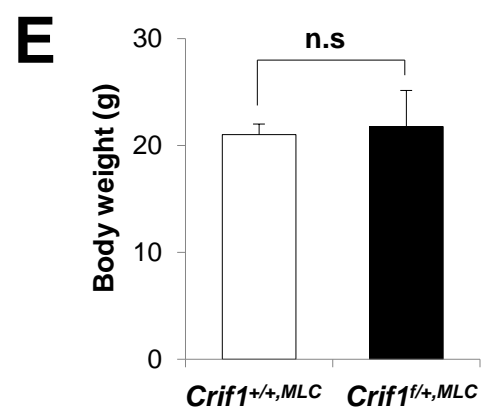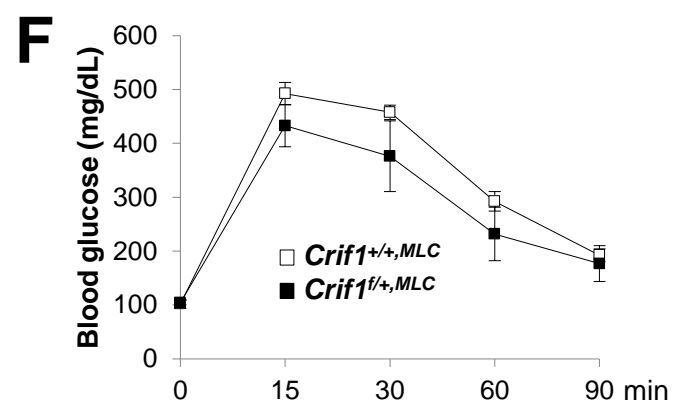

Supplement: Figure S10 — Metabolic phenotypes of liver specific- or skeletal muscle specific-Crif1 KO mice. (A and B) Body weight and daily food intake of control (Crif1+/+,Alb) and liver specific-Crif1 homozygous KO mice (Crif1f/f,Alb) fed a HFD diet for 8 weeks (n = 8). Values are means + SD. n.s, not significant. (C) IPGTT experiment with Crif1+/+,Alb and Crif1f/f,Alb mice injected with 1 g/kg glucose after 16 h of fasting (n = 8). Values are means ± SD. (D) Real time PCR with specific primers to measure gluconeogenic gene expression in the livers of fasted mice. (E) Body weight of control (Crif1+/+,MLC) and skeletal muscle-specific Crif1 haploinsufficientt mice (Crif1f/+,MLC) fed a NCD diet. Values are means + SD. n.s, not significant. (F) IPGTT experiment with Crif1f/+,MLC mice injected with 2 g/kg glucose after 16 h of fasting (n = 8). Values are means ± SD. (PDF) [file pgen.1003356.s010.pdf]
