# Supplementary material for: Crif1 Deficiency Reduces Adipose OXPHOS Capacity and Triggers Inflammation and Insulin Resistance in Mice
Source: PLoS Genet. 2013 Mar 14;9(3):e1003356. doi: 10.1371/journal.pgen.1003356 (PMC3597503; doi:10.1371/journal.pgen.1003356)
Supplement: Figure S5 — Gene expression profiles in 3T3-L1 adipocytes following silencing of Crif1 determined by a complementary DNA microarray. A microarray was performed using 3T3-L1 adipocytes treated with control or Crif1 siRNA with Agilent's DNA microarray Chip. Data were analyzed using the Feature Extraction and GeneSpring Software (Agilent Technologies). (PDF) [file pgen.1003356.s005.pdf]

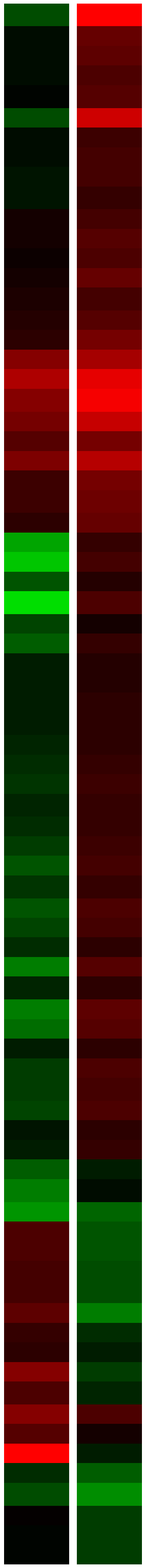

siCTR	si*Crif1*	Genbank	GeneSymbol	GeneName
4.121792	36.16663	NM_144548	IL23R	interleukin 23 receptor
40.50882	58.58208	NM_011612	TNFRSF9	tumor necrosis factor receptor superfamily, member 9
1961.249	2588.521	NM_009398	TNFAIP6	tumor necrosis factor alpha induced protein 6
28666.23	35917.62	NM_013654	CCL7	chemokine (C-C motif) ligand 7
24.32665	30.60764	NM_009401	TNFRSF8	tumor necrosis factor receptor superfamily, member 8
7.611051	27.08002	NM_009403	TNFSF8	tumor necrosis factor (ligand) superfamily, member 8
313044.8	352221.8	NM_019494	CXCL11	chemokine (C-X-C motif) ligand 11
340.7805	401.6685	NM_011332	CCL17	chemokine (C-C motif) ligand 17
784.6999	948.9369	NM_009399	TNFRSF11A	tumor necrosis factor receptor superfamily, member 11a
893.2378	992.3398	NM_145636	IL27	interleukin 27
22.12826	25.28546	NM_010552	IL17A	interleukin 17A
494.2479	620.9611	NM_009398	TNFAIP6	tumor necrosis factor alpha induced protein 6
59.17831	68.91479	NM_011331	CCL12	chemokine (C-C motif) ligand 12
1243.83	1646.707	NM_011330	CCL11	chemokine (C-C motif) ligand 11
194.0143	218.5462	NM_145837	IL17D	interleukin 17D
98.8333	120.3648	NM_010555	IL1R2	interleukin 1 receptor, type II
3470.772	3772.112	NM_134159	IL17RC	interleukin 17 receptor C
272.4725	304.6156	NM_028075	TNFRSF13C	tumor necrosis factor receptor superfamily, member 13c
413.7915	534.68024	NM_031168	IL6	interleukin 6
16.18136	32.31217	NM_183391	TNFSF18	tumor necrosis factor (ligand) superfamily, member 18
58.67183	93.43846	NM_010553	IL18RAP	interleukin 18 receptor accessory protein
971.2447	1121.058	NM_008364	IL1RAP	interleukin 1 receptor accessory protein
1455.233	2063.002	NM_010743	IL1RL1	interleukin 1 receptor-like 1
554.2708	702.7033	NM_001025602	IL1RL1	interleukin 1 receptor-like 1
692.054	856.0938	NM_009398	TNFAIP6	tumor necrosis factor alpha induced protein 6
49.0237	61.19808	NM_001039701	IL1RN	interleukin 1 receptor antagonist
21.90168	45.19971	NM_013563	IL2RG	interleukin 2 receptor, gamma chain
6.868854	18.9351	NM_001013412	CCL26	chemokine (C-C motif) ligand 26
23.93204	31.70892	NM_178258	IL22RA2	interleukin 22 receptor, alpha 2
5.921903	20.36962	NM_019568	CXCL14	chemokine (C-X-C motif) ligand 14
104.4863	127.2165	NM_021349	TNFRSF13B	tumor necrosis factor receptor superfamily, member 13b
23.35535	34.23926	NM_145834	IL17C	interleukin 17C
1655.112	1793.896	NM_008360	IL18	interleukin 18
**58167.016**	**81624.16**	**NM_011333**	**CCL2**	**chemokine (C-C motif) ligand 2**
79832.97	88935.83	NM_001172054	IL11RA1	interleukin 11 receptor, alpha chain 1
954.9624	1056.854	NM_029646	IL34	interleukin 34
19827.82	22730.69	NM_010560	IL6ST	interleukin 6 signal transducer
37.25387	44.44884	NM_028075	TNFRSF13C	tumor necrosis factor receptor superfamily, member 13c
90.76215	117.2686	NM_139299	IL31RA	interleukin 31 receptor A
1739.551	2058.738	NM_011614	TNFSF12	tumor necrosis factor (ligand) superfamily, member 12
**1978.869**	**2382.897**	**NM_013653**	**CCL5**	**chemokine (C-C motif) ligand 5**
45.11165	60.9423	NM_008764	TNFRSF11B	tumor necrosis factor receptor superfamily, member 11b
**1260.824**	**1926.372**	**NM_021274**	**CXCL10**	**chemokine (C-X-C motif) ligand 10**
5715.326	7169.517	NM_011338	CCL9	chemokine (C-C motif) ligand 9
685.4969	1091.775	NM_134437	IL17RD	interleukin 17 receptor D
2263.516	3224.157	NM_021443	CCL8	chemokine (C-C motif) ligand 8
304.5928	367.2768	NM_008353	IL12RB1	interleukin 12 receptor, beta 1
37.51998	75.69471	NM_009400	TNFRSF18	tumor necrosis factor receptor superfamily, member 18
37.8838	43.56515	NM_027206	TNFAIP8L2	tumor necrosis factor, alpha-induced protein 8-like 2
13.8947	28.78862	NM_080729	IL25	interleukin 25
1734.652	3219.236	NM_008369	IL3RA	interleukin 3 receptor, alpha chain
71.48267	80.16624	NM_177371	TNFSF15	tumor necrosis factor (ligand) superfamily, member 15
**43.57612**	**62.77047**	**NM_008599**	**CXCL9**	**chemokine (C-X-C motif) ligand 9**
30.23543	41.89107	NM_153077	IL1F10	interleukin 1 family, member 10
255.5504	379.3089	NM_001164724	IL33	interleukin 33
15516.67	16859.38	NM_009396	TNFAIP2	tumor necrosis factor, alpha-induced protein 2
1309.808	1477.902	NM_025566	TNFAIP8L1	tumor necrosis factor, alpha-induced protein 8-like 1
111.9911	133.0651	NM_178931	TNFRSF14	tumor necrosis factor receptor superfamily, member 14
40.64213	57.88874	NM_145856	IL17F	interleukin 17F
124.1442	142.8147	NM_008368	IL2RB	interleukin 2 receptor, beta chain
617.4527	348.2624	NM_008356	IL13RA2	interleukin 13 receptor, alpha 2
1066.871	587.4594	NM_001034031	IL17RE	interleukin 17 receptor E
2160.096	1276.128	NM_009139	CCL6	chemokine (C-C motif) ligand 6
101.98523	59.25557	NM_021283	IL4	interleukin 4
93.71742	40.71966	NM_019577	CCL24	chemokine (C-C motif) ligand 24
31.92887	22.55596	NM_008355	IL13	interleukin 13
91.77756	72.49704	NM_024290	TNFRSF23	tumor necrosis factor receptor superfamily, member 23
196.8808	81.45166	NM_178257	IL22RA1	interleukin 22 receptor, alpha 1
44.34467	28.77423	NM_010556	IL3	interleukin 3
1034.483	664.6325	NM_018866	CXCL13	chemokine (C-X-C motif) ligand 13
4273.788	3079.896	NM_008176	CXCL1	chemokine (C-X-C motif) ligand 1
335.2468	27.00682	NM_019508	IL17B	interleukin 17B
82.60403	65.24875	NM_010558	IL5	interleukin 5
31.3036	22.62042	NM_008348	IL10RA	interleukin 10 receptor, alpha
8953.882	6847.658	NM_020275	TNFRSF10B	tumor necrosis factor receptor superfamily, member 10b
339.0015	262.4593	NM_133990	IL13RA1	interleukin 13 receptor, alpha 1
5624.466	3258.674	NM_009138	CCL25	chemokine (C-C motif) ligand 25
