# Supplementary material for: Crif1 Deficiency Reduces Adipose OXPHOS Capacity and Triggers Inflammation and Insulin Resistance in Mice
Source: PLoS Genet. 2013 Mar 14;9(3):e1003356. doi: 10.1371/journal.pgen.1003356 (PMC3597503; doi:10.1371/journal.pgen.1003356)
Supplement: Table S1 — Summary of the phenotypes of adipocyte-specific Crif1 mutant mice under the control of the Fabp4-Cre or Adipoq-Cre promoters. The percentages represent residual levels in comparison to control mice.* The band intensities of Crif1 protein were determined by Western blot analysis using 6 male mice per group. ** Adipose mass was evaluated by totaling the weights of eWAT of 6 male mice per group. n.d; not determined. (DOCX) [file pgen.1003356.s011.docx]

**Table S1. Summary of the phenotypes of adipocyte-specific *Crif1* mutant mice under the control of the Fabp4-*Cre* or Adipoq-*Cre* promoters.**

| Promoters | Genotypes | Viability | Crif1 protein expression* | | Adipose mass** | | Inflammation |
| --- | --- | --- | --- | --- | --- | --- | --- |
|  |  |  | WAT | BAT | WAT | BAT |  |
| Fabp4-*Cre* | Control (*Crif1^+/+,Fabp4^*) | Viable | 100% | 100% | Normal | Normal | None |
|  | Heterozygous (*Crif1^f/+,Fabp4^*) | Viable | ~50% | ~100% | Normal | Normal | Moderate  (macrophage-predominant) |
|  | Homozygous (*Crif1^f/f,Fabp4^*) | Postnatally lethal at 3 weeks | n.d. | ~ 50%  (3 weeks after birth) | ~10% | ~30% | n.d. |
| Adipoq-*Cre* | Control (*Crif1^+/+,Adipoq^*) | Viable | 100% | 100% | Normal | Normal | None |
|  | Heterozygous (*Crif1^f/+,Adipoq^*) | Viable | 100% | 100% | Normal | Normal | None |
|  | Homozygous (*Crif1^f/f,Adipoq^*) | Viable | ~30% | ~20% | ~50% | ~80% | Moderate to severe  (macrophages and lymphocytes) |
