# Supplementary material for: Crif1 Deficiency Reduces Adipose OXPHOS Capacity and Triggers Inflammation and Insulin Resistance in Mice
Source: PLoS Genet. 2013 Mar 14;9(3):e1003356. doi: 10.1371/journal.pgen.1003356 (PMC3597503; doi:10.1371/journal.pgen.1003356)
Supplement: Table S2 — Sequence of primers used in real-time PCR. (DOCX) [file pgen.1003356.s012.docx]

**Table S2. Sequence of primers used in real-time PCR.**

| Gene name | 5’ Forward primer 3’ | 5’ Reverse primer 3’ |
| --- | --- | --- |
| *Crif1* | GAACGCTGGGAGAAAATTCA | ATAGTTCCTGGAAGCGAGCA |
| *Pparg* | ATCTTAACTGCCGGATCCAC | TGGTGATTTGTCCGTTGTCT |
| *Adiponectin* | TCTCCTGTTCCTCTTAATCCTGCC | CATCTCCTTTCTCTCCCTTCTCTCC |
| *Cd36* | TGCTGGAGCTGTTATTGGTG | TGGGTTTTGCACATCAAAGA |
| *Mcp1* | CCCAATGAGTAGGCTGGAGA | TCTGGACCCATTCCTTCTTG |
| *Ip10* | AAGTGCTGCCGTCATTTTCT | CCTATGGCCCTCATTCTCAC |
| *Rantes* | GTGCCCACGTCAAGGAGTAT | TCCTTCGAGTGACAAACACG |
| *Cd11c* | CAAAATCTCCAACCCATGCT | CACCACCAGGGTCTTCAAGT |
| *Tnfα* | CCCCAAAGGGATGAGAAGTT | CACTTGGTGGTTTGCTACGA |
| *Cd11b* | AAGGATTCAGCAAGCCAGAA | TAGCAGGAAAGATGGGATGG |
| *Arg1* | AAAGCTGGTCTGCTGGAAAA | ACAGACCGTGGGTTCTTCAC |
| *Cd68* | GCAGCACAGTGGACATTCAT | TTGCATTTCCACAGCAGAAG |
| *G6pase* | ACCCAGAAGACTGTGGATGG | TTCAGCTCTGGGATGACCTT |
| *Pck* | ATCATCTTTGGTGGCCGTAG | ATCTTGCCCTTGTGTTCTGC |
| *Gapdh* | CCAGTCGACTCGCTATCTCC | CGAGGAAAGAAAAAGCCAAC |
| *18s rRNA* | CTGGTTGATCCTGCCAGTAG | CGACCAAAGGAACCATAACT |
