# Supplementary material for: Crif1 Deficiency Reduces Adipose OXPHOS Capacity and Triggers Inflammation and Insulin Resistance in Mice
Source: PLoS Genet. 2013 Mar 14;9(3):e1003356. doi: 10.1371/journal.pgen.1003356 (PMC3597503; doi:10.1371/journal.pgen.1003356)
Supplement: Figure S8 — Phenotypic analysis and detection of Cre-recombinase activity in peritoneal macrophages from Crif1+/+,Fabp4 and Crif1f/+,Fabp4 mice. (A) Peritoneal macrophages were collected following intraperitoneal injection of thioglycollate into 6-week-old Crif1+/+,Fabp4 and Crif1f/+,Fabp4 mice. Expression of Crif1 and phenotypic markers for M1 and M2 macrophages were measured by real-time PCR using specific primers (n = 10). Values are means + SD. *p<0.05, n.s, not significant. (B) Schematic showing the loxp site in the Crif1 gene and the location of primers 1, 2, and 3. (C) PCR analysis of homologous recombination in genomic DNA extracted from WAT, BAT, and peritoneal macrophages isolated from 20-week-old control and Crif1f/+,Fabp4 mice. Genomic DNA for Cre recombinase was detected in WAT, BAT, and peritoneal macrophages (upper panel), but the recombination product was only present in WAT and BAT (lower panel). (PDF) [file pgen.1003356.s008.pdf]

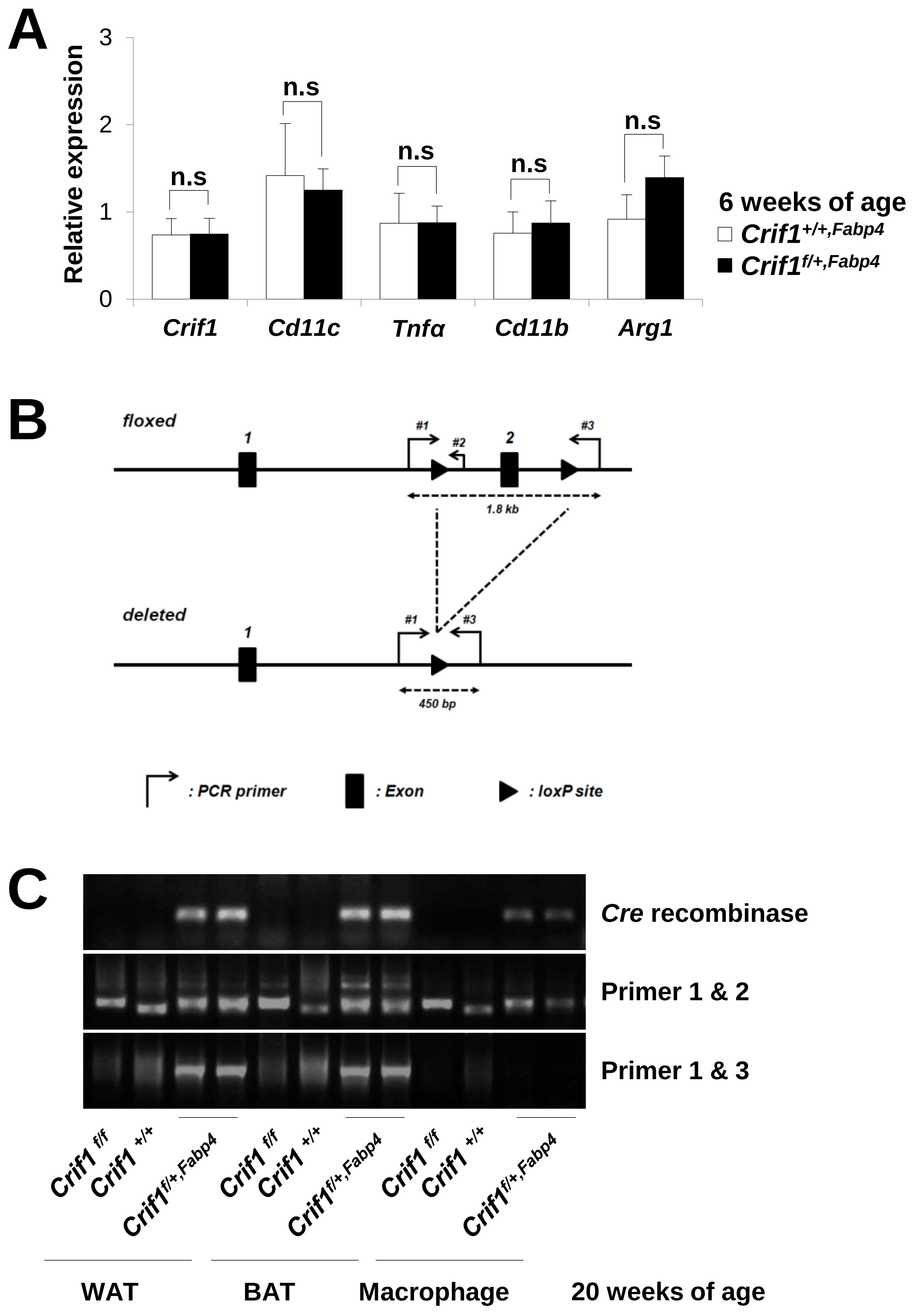
A
Relative expression
0
1
2
3
n.s
Crif1
Cd11c
Tnfα
Cd11b
Arg1
6 weeks of age
Crif1+/+,Fabp4
Crif1f/+,Fabp4
B
floxed
deleted
1
2
#1
#2
#3
1.8 kb
450 bp
: PCR primer
: Exon
: loxP site
C
Cre recombinase
Primer 1 & 2
Primer 1 & 3
Crif1 f/f
Crif1 +/+
Crif1f/+,Fabp4
WAT
BAT
Macrophage
20 weeks of age
